# Partner-Aware Prediction of Interacting Residues in Protein-Protein Complexes from Sequence Data

**DOI:** 10.1371/journal.pone.0029104

**Published:** 2011-12-14

**Authors:** Shandar Ahmad, Kenji Mizuguchi

**Affiliations:** National Institute of Biomedical Innovation, Osaka, Japan; University Of Oxford, United Kingdom

## Abstract

Computational prediction of residues that participate in protein-protein interactions is a difficult task, and state of the art methods have shown only limited success in this arena. One possible problem with these methods is that they try to predict interacting residues without incorporating information about the partner protein, although it is unclear how much partner information could enhance prediction performance. To address this issue, the two following comparisons are of crucial significance: (a) comparison between the predictability of inter-protein residue pairs, i.e., predicting exactly which residue pairs interact with each other given two protein sequences; this can be achieved by either combining conventional single-protein predictions or making predictions using a new model trained directly on the residue pairs, and the performance of these two approaches may be compared: (b) comparison between the predictability of the interacting residues in a single protein (irrespective of the partner residue or protein) from conventional methods and predictions converted from the pair-wise trained model. Using these two streams of training and validation procedures and employing similar two-stage neural networks, we showed that the models trained on pair-wise contacts outperformed the partner-unaware models in predicting both interacting pairs and interacting single-protein residues. Prediction performance decreased with the size of the conformational change upon complex formation; this trend is similar to docking, even though no structural information was used in our prediction. An example application that predicts two partner-specific interfaces of a protein was shown to be effective, highlighting the potential of the proposed approach. Finally, a preliminary attempt was made to score docking decoy poses using prediction of interacting residue pairs; this analysis produced an encouraging result.

## Introduction

Protein-protein interactions are crucial for almost all aspects of cellular dynamics in living systems [1], and an enormous amount of research has been conducted to reveal, understand and predict protein-protein interactions at species and cellular levels (e.g., [2]). Despite the central role of protein-protein interactions in the theme of life, a complete protein-protein interactome has not yet been deciphered, even for a small organism, and continuous efforts are being made to refine the available data [3]. Solving the three-dimensional structure of a protein complex can provide a detailed understanding of a specific protein-protein interaction (e.g., [4], [5]), but this type of technology becomes inaccessible on a genomic scale. Pair-wise associations between proteins can be inferred much more rapidly from high-throughput experiments, such as yeast two hybrid assays or mass spectrometry [6], but they cannot provide insights into the detailed mechanisms involved in such interactions, which is essential for therapeutic or biotechnological interventions. Therefore, a large number of putative interactions remain uncharacterized due to this technological gap.

Many protein structures have been solved (or can be modeled accurately), and the structure of a complex can, in principle, be obtained by docking its constituents. However, protein-protein complexes are formed as a result of numerous interactions at tertiary and quaternary structure levels; therefore, the task of building a complex from these individual units represents a considerable challenge. Prediction of interacting regions between a pair of proteins is a step toward elucidating the final mode of interaction between the proteins. For this purpose, a sequence-based approach is likely to be more convenient and faster than structure-based methods because of the lower dimensionality of the input data and the abundant sequence information. The underlying principle behind this approach has been to identify a relationship between readily computable sequence features (e.g., residue type) and the quantities that characterize the interaction (e.g., residue contact or the change in the free energy of the association). Once a relationship has been established, novel interactions can be detected via these features. A number of studies have been performed attempting to model this relationship (e.g., [7], [8]). Researchers have also endeavored to distinguish physical interactions from random associations [9], transient interactions from obligatory complexes [10], crystal packing from oligomerization [11] and specificity from affinity and promiscuity [12].

Prediction-oriented studies generally address one of the two following problems: (a) given a set of proteins, to determine which pairs interact with each other and (b) given a single-protein sequence (or structure), to determine sequence (or structural) regions that would interact with *any* other protein. Both types of studies have relied on a variety of sequence, structural or other data sources, such as microarray data [9], protein structures [13], [14], [15], conservation of interaction sites [16], [17], clustering of conserved residues [18], co-evolution statistics [19] and codon usage [20]. A variety of computational techniques have been employed to utilize this information, including neural networks [21], [22], [23], [24], [25], support vector machines [26], [27], [28], [29], [30], [31], [32], random forests [33] and Bayesian techniques [34], [35], [36], [37], [38].

In this study, we are concerned with the second problem, and we aim to predict interacting residues from sequence information alone. However, we intend to go beyond the current regime of predicting residues that would interact with any protein; instead, we aim to identify interacting residue pairs between two specific proteins. A more specific objective of the current study was to assess whether the performance of sequence-based prediction of interacting residues can be improved by training models on interacting residue pairs with knowledge of the interacting partner protein. To answer this question, we trained a two-stage neural network model on a data set composed of interacting residue pairs from known protein complexes; next, we trained a similar two-stage model on a data set of single residues extracted from the same data source (without using any pairing information). The performance of the models trained either on residue pairs or on single residues was compared by predicting both the interacting residue pairs and the interacting single residues. The results showed that the models trained on residue pairs outperformed those trained on single residues on both accounts. Similar to docking, the prediction performance was anti-correlated with the size of the conformational change that was induced upon complex formation.

Furthermore, we carried out a preliminary assessment regarding the possibility of using this method to predict multiple interfaces of a protein with different partners, and we obtained an encouraging result. We also made a preliminary attempt to use the proposed method as a scoring function for protein-protein docking, and we showed that our simple procedure was competitive against a more sophisticated structure-based approach.

## Methods

### Data Set

The protein-protein docking benchmark data set (version 3.0) compiled by Hwang et al. [39], which is abbreviated DBD3.0 in this work, was used throughout this study. We chose this data set because it was systematically curated and included protein complexes (each consisting of a “ligand” and a “receptor”) for which the unbound structures of both the ligand and the receptor were available, thus allowing us to analyze our results in the context of conformational changes. Furthermore, the data set also provided pre-computed ranked decoy sets, and we utilized this resource to score the docking decoys (see below).

This data set contains 124 complexes, and we used only the bound structures for the current study. The authors constructed DBD3.0 such that no two complexes shared an identical set of families defined in Structural Classification of Proteins (SCOP) [40] (see [39] for details). Thus, the data set was non-redundant, but the redundancy was defined somewhat differently from the typical sequence-based prediction methods. We evaluated sequence-level redundancy using BLASTCLUST [41] and confirmed that no two complexes shared more than 30% sequence identity in both the ligand and the receptor chains, i.e., at least one protein in the pair was unique.

**Figure 1:**
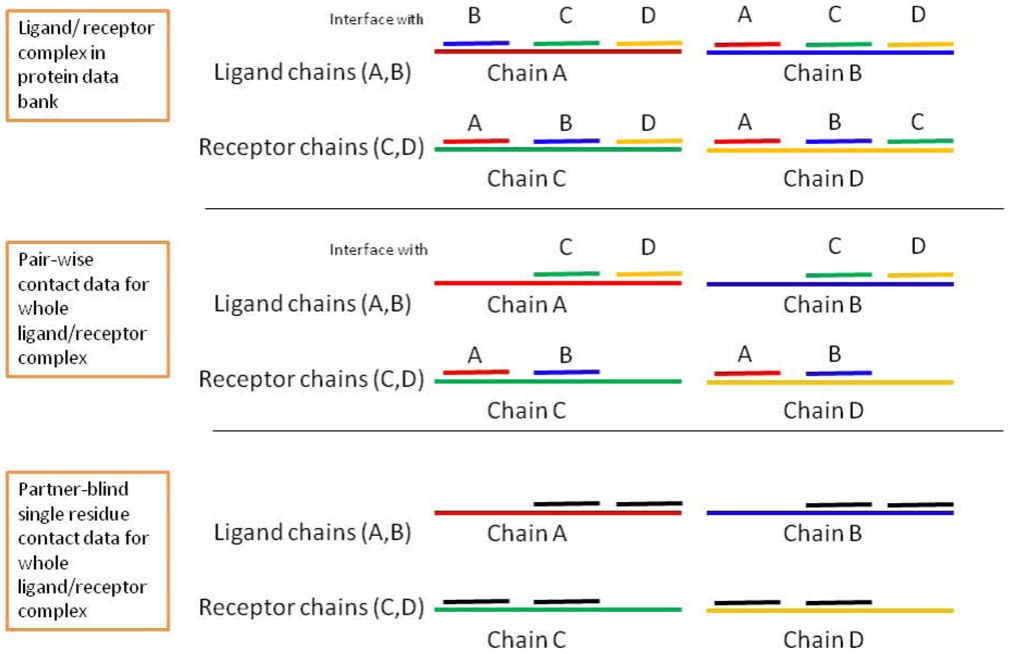
Residue pair and single-residue contact data preparation from ligand/receptor complexes (an example with a dimeric ligand complexed with a dimeric receptor is shown here). For each of the 124 complexes, the data sets were prepared by pairing residues from the ligand and receptor chains for a pair-wise prediction (as shown in the second part of the illustration). Single-residue data were also prepared for the whole complex. However, the residues were not encoded as pairs; they were taken from individual chains and partner information was discarded, and the contact data for all the chains were pooled together to obtain whole-complex data. In one training cycle, the contact and feature data from all but one of the complexes were used for training, and the left-out complex data were then used to evaluate prediction performance. Performance scores were calculated for one complex in one training cycle. The obtained set of 124 scores was then averaged to obtain an overall performance score.

To achieve unbiased training and evaluation, we adopted the procedure described in Figure 1. In DBD3.0, a ligand (or receptor) may consist of more than one protein chain. Each chain was treated separately, but only the interactions between the ligand and receptor were considered (thus, interactions within the ligand or receptor chains were ignored). Data were pooled for all the chains from both the ligand and the receptor to produce a single performance metric for each complex. For example, if there were m_1_ and m_2_ residues in the two chains of a ligand and n_1_ and n_2_ residues in the two chains of a receptor, a total of (m_1_+m_2_)*(n_1_+n_2_) residue pairs were considered, and an attempt was made to classify them as either interacting or non-interacting. Likewise, a total of m_1_+m_2_+n_1_+n_2_ residues were considered in predicting the interacting residues in a single chain, and the results were pooled together.

### Interface residue definition

A pair of residues from different chains of proteins was labeled as belonging to the positive class (binding) if the distance between any atom of one residue and any atom of the other was less than or equal to 6.0 Å. This distance cutoff has been used in other studies [42]. Contacts within multiple chains of a single ligand or a receptor were ignored, as illustrated in Figure 1.

### Propensity scores

As in our previous studies, we used propensity scores for single-residue contacts as a ratio between the relative number of that residue type in the interface and the relative number of residues of any type in the interface [43], [44]. This definition was extended to pair-wise contacts in a similar way. Specifically, the interface propensity of a residue pair with indices *i* and *j* (where *i* and *j* have values from 1 to 20) is given by the following equation:
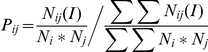
where *N_i_* refers to the number of residues of residue type *i* (e.g., Arg) and *N_ij_(I)* is the number of contacting residue pairs identified by indices *i* and *j* in the interface. Summation was performed over all the residue pair types.

The statistical significance of the overrepresentation of certain residue pairs was assessed using a chi-squared test comparing the observed and expected numbers of contacts for each residue pair. The observed number of contacts (*O_ij_*) was obtained for the entire set of proteins, and the expected number of contacts (*E_ij_*) between amino acids *i* and *j* in one protein complex was computed using the following equation:
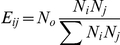
where *N_o_* is the total number of observed contacts among all the residue pairs and the subscript *i* and *j* are used for ligand and receptor residues respectively. The expected number was computed for each complex separately, and the numbers were then added to obtain a final value. This expected number of contacts was compared with the observed number of contacts for each pair of residue types, and a chi-squared value was computed using the following formula:
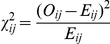
and compared with the values from the standard table with a single degree of freedom.

### Interacting residue-pair predictions

Predictions were performed by identifying a trained model that could relate a set of sequence features from a pair of target residues and their sequence neighbors to their contact state (binding or non-binding, as defined above). The sequence feature set refers to (a) sparsely encoded sequence features, such as those used in classical works on secondary structure prediction [45]; (b) position-specific scoring matrix (PSSM)-based features that are similar to our previous studies [43]; and (c) a combination of (a) and (b). Sparsely encoded sequence features simply represent each amino acid by a 20-dimensional vector, in which all but one of the dimensions are set to zero and one dimension corresponding to that residue type is set to 1. On the other hand, PSSM-based encoding represents each residue by the log-odd frequency of occurrence of the 20 residue types in an alignment at the target residue position (alignment column). The PSSM was obtained by running 3 iterations of PSIBLAST [41] using the default parameters against the NCBI NR database. Typically, an *m*-residue window is used for PSSM encoded features, and an *n*-residue window is employed for a sparsely encoded residue, where m and n range from 0 to the maximum window size (7 residues in this study). The resulting *m+n* features from each residue at position *i* and *j* in two contacting chains were concatenated in both orders (*i*,*j* and *j*,*i*), which created two patterns for the neural network inputs with identical target outputs. Using the features in both orders allowed the neural network to automatically learn that the pattern vector was independent of the order of the residues in the pair. Accordingly, model performance was evaluated by making a prediction for each residue pair in both orders and using the average of the two as the final score. The target output for the neural network was set to 0 or 1, which corresponded to the negative and positive class labels as defined above. The neural networks returned a real number between 0 and 1, which was converted to a class label using the procedure described in the performance evaluation section.

In the first stage of prediction, 24 independent neural network models were trained and assessed by leave-one-out cross validation. The models were allowed to learn for a fixed number of cycles without using information from the protein that was left out. The prediction performance for each left-out protein was computed from a model trained in the absence of this protein, and the scores were averaged to obtain an overall performance score. The first stage neural network models differed from each other in terms of the following characteristics:

- Feature sets: Different window sizes were used for the sparse and PSSM-encoded environments of the residue pairs that ranged from 0 to 3 residue neighbors (*n* sequence neighbors from the N- and C- terminal position leads to a *(2n+1)* residue window, which results in values of 0 to 7). Because there were 5 such possibilities for each of the sparse and PSSM-encoded features, a total of 5×5 possible combinations remained. Of these remaining combinations, 1 (0 for PSSM and 0 for sparse encoding) was a featureless representation that was discarded; this left 24 independent models. Terminal positions where N- and C-terminal residues are not present and hence pattern vectors could not be created have been excluded from the training/validation cycle data sets.
- Negative data sampling: In each of the 24 models, training was performed by sampling negative data because negative class data (non interacting residue pairs) were approximately 500 times more prevalent than positive class data (interacting residue pairs). To overcome the training difficulties caused by this imbalance, only 2% (or 1,000, whichever was smaller) of the randomly selected negative data points were used for training. All the positive data points were retained. No sampling was performed for the cross-validation (blind) data, and the reported performance measures were based on the real data. The 2% residue-pair data corresponded to approximately 14% (square root of 0.02) of the data from each of the two interacting proteins; therefore, the single-protein training models sampled 14% of the negative data. Each of the 24 models was trained on different random samples, which allowed for noise cancelation between models in the stage 2 predictions.

**Figure 2:**
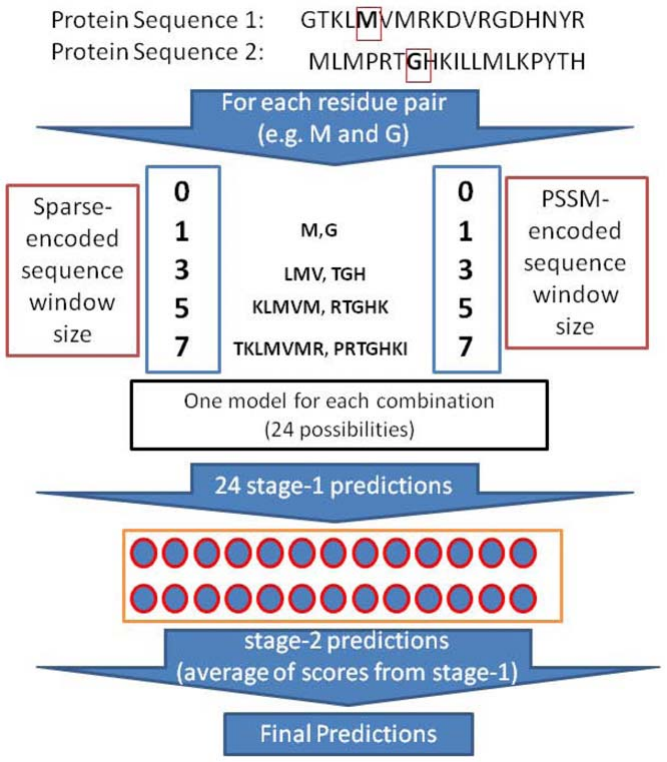
Overall prediction of interacting pairs of residues in two stages.

In the second stage, the first stage predictions produced by the 24 neural networks were averaged to obtain the final prediction (see Figure 2).

### Pair prediction to single-chain predictions

To convert a pair-wise prediction score to a conventional interface residue prediction in single proteins, pair-wise prediction was first performed as described above. For each residue in each protein, we next assigned the highest pair-wise score that involved that residue. Thus, all the residues in all the chains were assigned a real value score, which is analogous to a single-chain prediction score, and all performance parameters were computed from these scores.

### Single-chain to pair-wise predictions for benchmarking

The currently available methods for predicting protein interaction sites return a single score for each residue in each protein chain, irrespective of its partner. To obtain a pair-wise prediction score that could be used in a comparison, we performed single-chain predictions for the individual chains and then calculated the pair-wise score of a residue pair by averaging the individual scores of the two residues in the pair.

### Performance measure

All the prediction models were trained to return a real number between 0 and 1, and the desired class labels were binary (1 for interface residues and 0 for non-interface residues). The output real numbers were converted into a class prediction by selecting different thresholds (thereby changing the number of residues that were predicted to be in the interface), and performance was evaluated. At a given threshold, any correctly predicted interface residues were designated as true positives (and their counts were denoted TP), whereas any correctly predicted non-interface residues were designated as true negatives (TN). Similarly, false positives (FP) and false negatives (FN) were residues that were wrongly predicted to be in the positive or negative class, respectively. For each threshold, the sensitivity (also called recall), precision and specificity of the model were defined as follows:
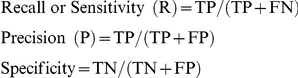
To consider both recall and precision, the F1-measure (the harmonic mean of precision and recall) was defined as follows:
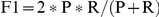
Because the balance between these scores changes with the threshold, a single performance measure was required to compare the performance of the various models. The two following measures are common: (i) the area under the receiver operating characteristic (ROC) curve, or AUC, where the ROC is a plot of the recall against (1-specificity), and this measure considers an entire range of threshold values; and (ii) a set of precision, recall and F1 at the best performing threshold, at which F1 takes the highest value. For most comparisons, we used the AUC as the main performance measure, and in some cases, we provide recall, precision and F1 scores as an additional reference in the supplementary materials.

In our leave-one-out scenario, all the performance scores were computed for each (left-out) protein complex and averaged (over 124 values) to obtain an overall estimate.

## Results and Discussion

### Propensity of pair-wise versus single-protein-residue preferences

**Figure 3:**
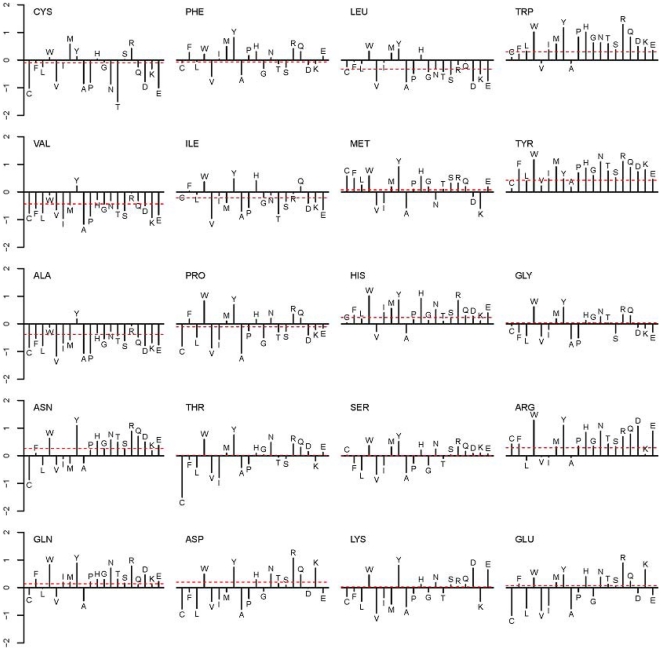
Sequence-based residue-pair contact propensities (natural logarithmic values) in a protein-protein interface. Each plot corresponds to interface propensity of a residue with all of the 20 possible partner residues. Single residue propensity values for the target residue are shown by a horizontal dashed line. See Table 1 for comments and Table S1 for additional details.

Table 1 shows the propensity scores of the 10 most preferred and the 10 most excluded residue pairs in the protein-protein interface (Table S1 provides complete details). The graphical pairing propensities of all the combinations are shown in Figure 3. Because our primary goal was to provide a comparison between single-residue preferences and pairing preferences, the propensities computed for the single residues to be in the interface are shown in the plot as a reference. The following observations were made from these results:

**Table 1:** The most significant contact occurrences in protein-protein interfaces derived from protein-protein complexes.

Pair(i–j)	Counts(N_i_*N_j_)	Propensity	Observed contacts(O_ij_)	Expected contactsE_ij_ = (N_i_*N_j_)/Σ(N_i_*N_j_)	Chi-squareχ^2^ = (O_ij_−E_ij_)^2^/O_ij_	p-value(Chi-square test)
**Enriched pairs**						
D-R	38053	2.9	161	55.3	202.2	6.85E-46
R-Y	27214	3.0	119	39.5	159.8	1.28E-36
N-Y	27275	3.0	118	39.6	155.1	1.36E-35
E-R	43020	2.4	152	62.5	128.2	1.01E-29
R-W	10880	3.6	57	15.8	107.4	3.67E-25
N-R	29478	2.4	104	42.8	87.4	8.80E-21
K-Y	38155	2.2	124	55.4	84.9	3.22E-20
D-K	53701	2.0	158	78.0	82.0	1.34E-19
E-K	61956	1.9	172	90.0	74.7	5.42E-18
W-Y	10501	3.2	49	15.3	74.7	5.60E-18
**Excluded pairs**						
A-V	74917	0.3	34	108.8	51.4	7.36E-13
A-L	90550	0.5	60	131.5	38.9	4.46E-10
L-V	94746	0.5	65	137.6	38.3	5.99E-10
K-V	69074	0.4	40	100.3	36.3	1.71E-09
E-L	84917	0.5	59	123.3	33.6	6.87E-09
E-V	70674	0.4	45	102.7	32.4	1.26E-08
I-V	57344	0.4	32	83.3	31.6	1.90E-08
A-P	49550	0.3	25	72.0	30.7	3.08E-08
D-L	74253	0.5	51	107.9	30.0	4.38E-08
S-V	81876	0.5	61	118.9	28.2	1.08E-07

The residue pairs presenting most significant p-values (top 10 from the favored and excluded categories each) are listed here. It should be noted that the data were derived from all residues of the complex, and that the surface propensity of the residues is implicitly included. Near absence of the hydrophobic residues in the top scoring pairs highlights the fact that from a purely sequence point of view hydrophobic pairs may not be the best interface candidates. However, if only the surface residues were considered (using structure information), situation might be different.

1. Several residues appeared to be highly preferred with one partner but highly excluded with another; e.g., Pro paired with Trp with a high propensity (2.3), but its pairing with Ala was one of the most excluded parings. Similarly, Phe paired with Tyr with a high propensity, but its pairing with Val was highly excluded from the interface. This observation justifies the pair-wise and partner-aware prediction that we aim to make.
2. Some residues showed less specific contact preferences than others; e.g., Tyr had a high propensity to pair with several residues, such as Arg, Phe, and Trp, which suggests a general preference for Tyr to be in the interface. Tyr can easily accommodate itself in both the loop and the strand regions of antibody complementarity determining regions (CDRs). Together with its amphiphilic nature, this often results in over-representation of Tyr in CDRs [46]. Although this reference addresses CDRs, similar interaction preferences of Tyr appeared in non-antibody complexes, as indicated in the results of this study.
3. Hydrophobic residues are not always preferred in the interface; this is evident because some pairs, such as Met-Val and Ala-Val, were excluded from the interface. Apparently, this result is in contrast to the observation of predominantly hydrophobic interactions contributing to folding and intra-chain residue-pair contacts in proteins, well documented in previous studies (e.g., [47], [48]). However, this apparent discrepancy must be interpreted in the context of the current propensity values being derived from sequences and considering that they implicitly include the surface propensity of the residues. Because many hydrophobic residues lie in the buried regions of a protein, the absence of a high propensity for hydrophobic residues only implies that these residues may naturally prefer the protein core to a protein-protein interface. An exposed hydrophobic residue may become preferred in the interface, which is an issue that we did not examine because we were interested in the sequence determinants of the interface residues. However, the propensities of the single residues within the surface have been examined by other researchers and can thus be referred to for comparison [49].
4. Electrostatic forces are also important because similarly charged Lys-Lys pairs were excluded, and oppositely charged Arg-Asp pairs were preferred. Interestingly, Arg did not appear in the excluded residue pair list except with a couple of partners with low statistical significance. On the other hand Lys did not always appear in the preferred list, which suggests that these residues play different roles, despite having identical charges. Arg has a higher propensity than Lys for the interface of protein-ligand and protein-nucleic acid complexes [43], [44]. This may be attributed to a number of structural and chemical attributes of Arg in contrast to Lys. For example, Arg can form a larger number of hydrogen bonds than Lys. Arg also exhibits pseudo-aromatic behavior due to the planar nature of its π-electron system [46].

Despite a clear sign of recognition that is promoted by the individual residue pairs, interface regions cannot be identified by simply locating complementary residue pairs because there are so many possible combinations and because sequence and structural neighbors are likely to constrain the actual population of interface residue pairs. The best estimates of these biases can be made by trying to predict the interface and then examining the prediction performance obtained from various feature sets. Therefore, we used a range of sequence windows encoded by residue identities and the evolutionary profile of each position to predict interface residue pairs from all the possible pairs of two proteins. The results are discussed in the following sections.

### Prediction performance

To obtain a thorough analysis, four types of predictions were compared. First, the models that were trained on residue pairs were used to estimate the performance of the left-out complex in a leave-one-out cross-validation regime. These pair-wise predictions were then converted to single-residue predictions by assigning the highest score of the pairs in which a given residue was involved.

Conversely, two prediction performance scores were obtained from the models that were trained on single proteins in a similar manner. The pair-wise scores were obtained by simply averaging the scores of the two residues in a pair. Thus, the ability to predict interacting pairs and single residues can be compared for the two sets of models, i.e., the models trained on pairs and those that were trained on single residues.

Table 2 summarizes all the performance scores measured by the AUC. The results of the pair-wise models for each protein are provided in Table S2. Protein-wise comparison of performance in the two prediction models with detailed ROC plots are shown in Figures S1 (prediction of residue pairs) and S2 (prediction of single residues).

**Table 2:** Comparison between the performance levels of the various models.

Prediction model	Prediction of residue pairs (AUC in %)			Prediction of interacting residues (AUC in %)		
	Paired training	Unpaired training	p-value	Paired training	Unpaired training	p-value
Leave-one-out (Stage 2)	72.9	71.0	0.0023	66.1	63.8	0.0062
Leave-one-out (Stage 1)	67.9	63.4	4.2e-6	64.6	59.6	6.0e-9
PSIVER*	-	62.8	-	-	57.5	-
SPPIDER*	-	58.4	-	-	54.1	-

The results from the current model are based on a 7-residue window from the protein and contain information from the sequence PSSM and global amino acid composition of the protein (for stage 2 models, the predictions from all window sizes from 1 to 7 were averaged). The p-values were computed by taking protein-wise performance scores and applying the paired Student's t-test over a set of values in the two models being compared.

*These online predictions (PSIVER and SPPIDER) are based on a single model and are optimized for binding site definitions and data sets that are different from those used in this study. Although our performance appears to be higher than those of these web servers, the choice of data sets, contact definitions and performance evaluation method used were not extensively examined because the main objective of this work was to establish the point that was made in the top two rows of this table. The performance scores from the online web servers are provided only as a record (see also the results and discussion).

The overall results can be summarized as follows.

### Models trained on residue pairs outperform the corresponding single-protein models

The first two rows of Table 2 show all four of the performance scores for the models that were trained on DBD3.0. The performances of the models trained on residue pairs were 72.9% and 66.1% for the residue pair and the single-residue predictions, respectively. The corresponding performances of the models trained on single residues were 71.0% and 63.8%, respectively. The performance of the pair-wise models was higher with respect to predicting both single residues and residue pairs, and the differences were statistically significant.

**Figure 4:**
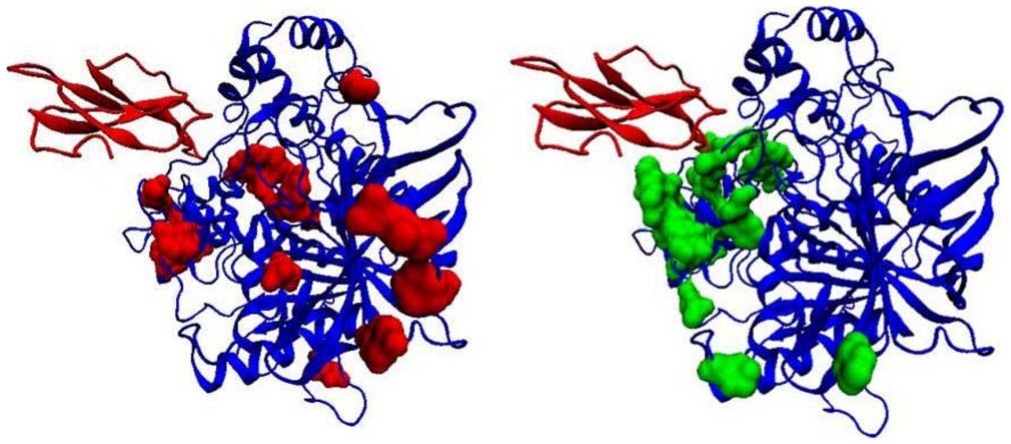
Binding site predictions mapped to the three-dimensional structure of Acetylcholinesterase in complex with Toxin F-VII Fasciculin-2 (PDB ID: 1MAH, chains A and F respectively in red and blue color cartoons). The left and right images were drawn from the top-scoring 20 predictions from single-protein trained models (solid red) and pair-wise trained models (solid green), respectively. Many false positive cases observed in the single-protein trained model were eliminated in the pair-wise model. (The false positive rate in the selected 20 residues is 75% and 50% with an overall AUC of ROC being 60% and 82%, respectively. Predictions are made from the models trained by excluding this complex from the training data.)

A typical example of the partner-unaware and pair-wise, partner-aware predictions is shown in Figure 4 using Acetylcholinesterase in complex with Toxin F-VII Fasciculin-2 (PDB ID: 1MAH). The predictions made from a model that was trained on single residues produced a number of false positives in the top scoring residues. This number significantly decreased after partner information was introduced by means of a pair-wise model. Quantitatively, the proportions of true positives that were in the top 20 positions in the two cases were 25% and 50%, respectively, showing a net improvement by 25 percentage points in this particular example. Presumably, the false positives were filtered out because the partner protein did not contain complementary residues for the candidates that were detected in the single-protein model.

### Two-stage models are significantly better than stage 1 models

Although most of the comparisons in this study were based on the final stage 2 model, we examined the performance of the stage 1 models in comparison to the final model. In general, the pair-wise model performance was approximately 2–6 percentage points higher than the first-stage model (Table 2). Similarly, the single-chain prediction models showed an improvement of 4–7 percentage points. Because the two-stage model essentially averages multiple predictions from closely related feature subsets, we believe that this improvement was caused by noise reduction because only the residues that showed high scores in all (or most) of the models were given high scores in the second stage. Because most of the published methods for predicting protein-protein interactions are based on single-stage computational models, they may benefit by employing this two-stage approach.

### Comparison with SPPIDER and PSIVER

In the last two rows of Table 2, we show the ability of two public web servers (SPPIDER [8] and PSIVER [34]) to predict single interacting residues. We also converted their prediction results into residue pair scores in the same way as described above.

While the prediction performance of our method was based on the leave-one-out cross-validation results and was therefore based on 124 models, the online web servers used a single model for all predictions. Furthermore, data redundancy, the definition of contacts and the performance evaluation method used were all different in the different studies, which made it rather difficult to directly compare their performances. For example, SPPIDER defines contacts by building a consensus over a number of similar instances in which a residue position occurs, thereby significantly enriching the number of positive class data points. This leads to a relatively large number of positive predictions (we observed that 3% of all the residues produced the maximum binding score). In our calculations, many of these contact predictions were flagged as false positives; however, according to the SPPIDER definition, these could be considered true positive cases. In the current study, we also discarded contacts within the chains of a single ligand or receptor (as illustrated in Figure 1). Therefore, although the performance of our models appears to be higher than that of the previously published methods, we do not claim that they provide a more accurate result; this claim would require more rigorous examination using common data sets, and various definitions of contacts would be necessary.

### Comparison with *PIPE Sites*

While this work was being completed, a related study was published [50]. In this publication, the authors reported the development of the web server *PIPE Sites*, which predicts interacting regions in a pair of protein sequences. The method essentially pairs protein sub-sequences at various window sizes and scans a database of known protein-protein interactions for their co-occurrence. Their prediction method does not use any training, and it is based on direct comparison. The method was benchmarked by measuring the overlap between the predicted paired regions and the pair of sequence regions annotated as interacting domains in a database [51]. We note that although the *PIPE Site* method is likely promising and useful, it addresses the problem of protein-protein interactions at a different level, as it detects relatively longer interacting regions. Because the *PIPE Site* predictions at the residue-pair level are unavailable, we were unable to perform even a rough quantitative comparison with our method.

### Protein-wise performance comparison

We next analyzed the protein-wise performance of our final stage 2 model and made the following observations.

### Complexes with large conformational changes are difficult to predict, although no structures are used in the prediction

**Figure 5:**
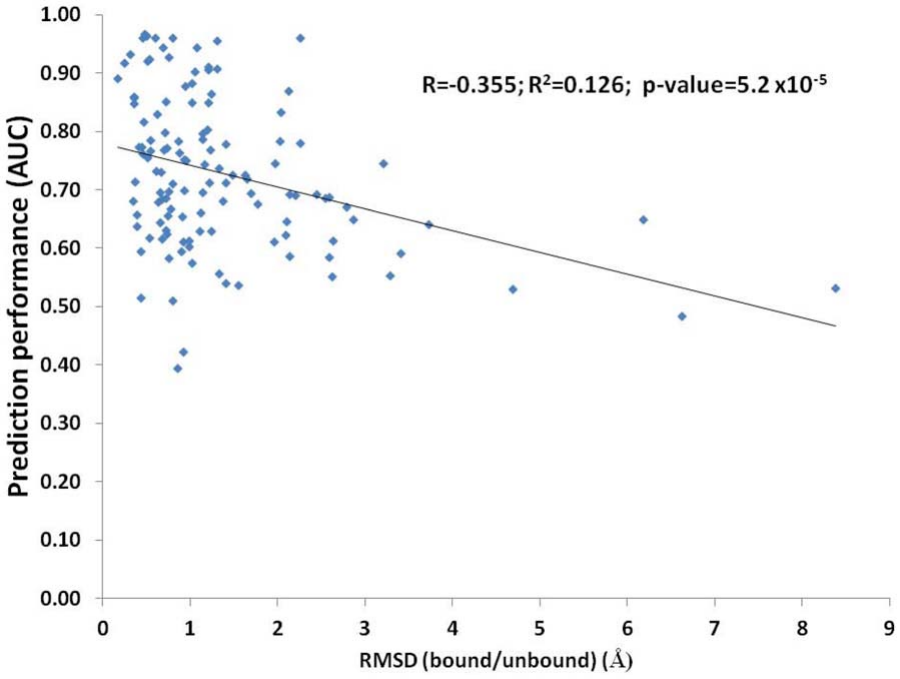
Relationship between prediction performance and the RMSD between bound and unbound complexes. Even though there are few data points in high RMSD category making the statistical point only suggestive in nature, poorer prediction performance for complexes undergoing large conformational change is consistent with the arguments in the discussion.

The creators of DBD3.0 [39] investigated the degree of difficulty in predicting docked complexes from unbound structures; they defined the three levels of difficulty involved as rigid body, medium and highly difficult complexes. Because this classification was based on structural considerations, i.e., the conformational changes that occur upon complex formation, it would be interesting to determine whether the pair-wise predictions derived purely from sequence features also follow the same pattern of difficulty levels. In Table 3, we summarize the performance of the pair-wise prediction results with respect to the three categories. To provide a more detailed view of this summary, we plotted the performance scores as a function of the root mean square deviation (RMSD) of the conformational change on complex formation in Figure 5. As shown in Table 3, the residue pairs in difficult class complexes were predicted to have scores that were 11.4 percentage points lower on average than the rigid body cases. An alternative demonstration of this result is the negative correlation (R = −0.355) that exists between the RMSD of the bound/unbound structure pairs and prediction performance (Figure 5). Therefore, we conclude that the structural changes introduced by complex formation are a challenge that must be resolved for both structure-based docking and sequence-based predictions; we presume that this challenge exists because the long-range intra-chain cooperativity of the interacting residues could not be learned by the prediction models.

**Table 3:** Performance of pair-wise predictions grouped by reported difficulty level in structure-based predictions and functional class.

Classification	Number of complexes	AverageAUC (%)
**Conformational** **Change**		
Rigid-body	88	75.1
Medium	19	70.6
Difficult	17	63.7
**Functional** **Class**		
Enzymes/inhibitor or Enzyme/substrate	35	74.2
Antibody/Antigen complex	25	90.0
Others (unclassified)	64	65.5

Complete data for the performance of each protein complex and other measures of performance (e.g., precision, recall and F-measure) are provided in Table S1. The classification was taken from the original curators [39].

### Performance grouped by the functional class of a complex

To analyze the variation of prediction performance, based on the functional class of a complex, we used the same functional classification provided by the creators of DBD3.0 [39]. Antibody/antigen complexes in that work were divided into two groups based on the availability of unbound structure. Since that classification is irrelevant for our sequence-based predictions, we merged them into a single category called Antibody/Antigen complexes. The last part of Table 3 shows the average performance of residue pair prediction in the three groups based on this classification. We observed a clear pattern that suggested the functional categories of Antibody/Antigen complexes to be the best predicted group of protein-protein complexes. This high prediction performance was produced potentially because antibody-antigen complexes may utilize some common patterns of interacting residue pairs, enabling these patterns to be detected by models trained on other complexes within these functional categories. The behavior of enzymes and their substrates and inhibitors is close to the overall average. The lowest performance was observed in the unclassified group, which was designated “others.” It is expected that for efficient prediction, several members of the same functional class should be present in the training data; the “others” category presumably consisted of several different functional protein classes, and these classes were not well represented in the data. Improvement of the annotation of complexes and enrichment of the data with samples from each functional class will likely improve performance for these cases.

### Application to detect partner-specific interface residues

**Figure 6:**
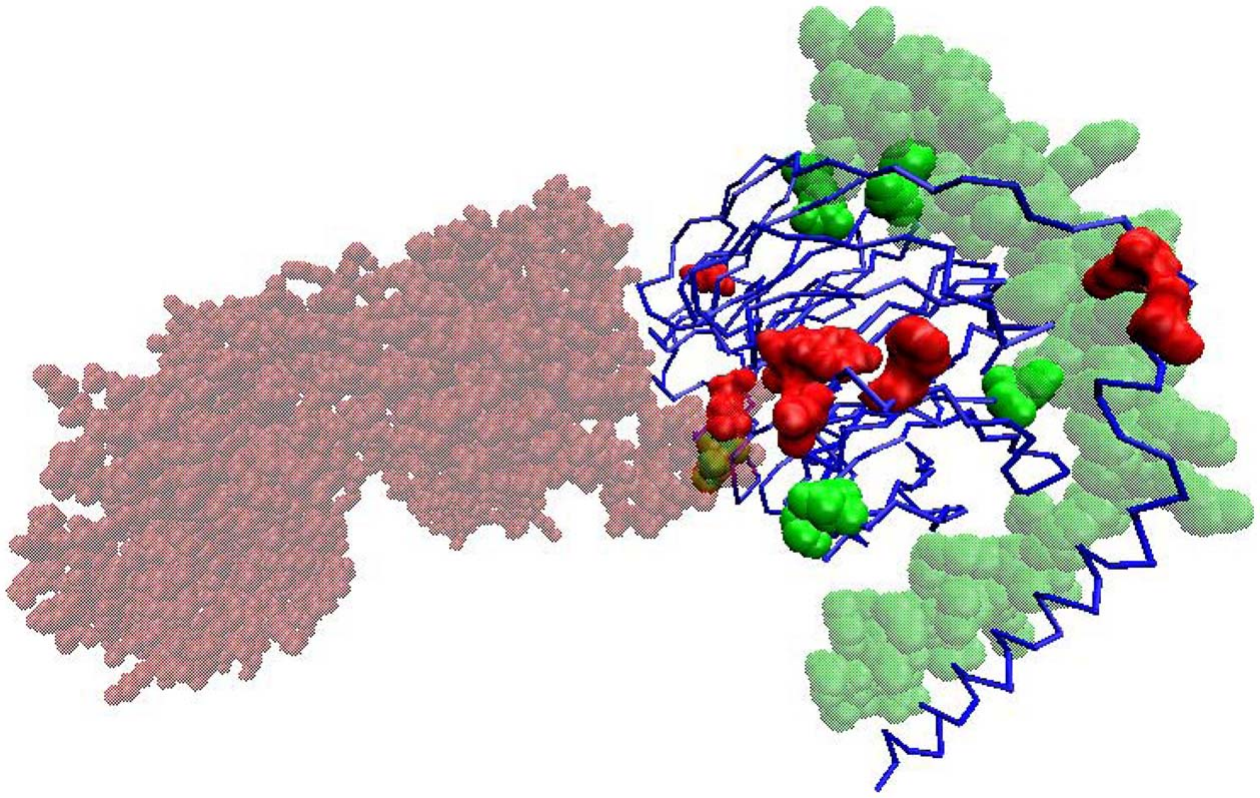
Partner-specific prediction of two interfaces for the beta subunit of the guanine nucleotide binding protein (transducin beta chain 1) (PDB ID: 3PSC, chain B is shown as the blue cartoon). The two partners are shown in transparent colors (chain A, which is the beta-adrenergic receptor kinase-1, is shown in red, and chain G, which is the guanine nucleotide binding protein's gamma-2 subunit, is shown in green). Predictions from the pair-wise model for each partner chain were converted into single chain predictions and displayed on chain B. Common binding sites, predicted with both partners, were removed and residues exclusively predicted with each partner are shown in the corresponding partner color. Out of the 30 top-scoring residues after removing common predictions, most residues have been assigned to the correct partner.

Previous sequence-based predictors of protein-interaction sites have aimed to detect interface residues without considering partner proteins. One significant application of the current method is that it can be used to distinguish between partner-specific interfaces of proteins that interact with more than one protein. Here, we examined a few multi-chain complexes from the Protein Data Bank (PDB) [52] that were not included in DBD3.0. One such example was the transducin protein beta chain 1 (PDB ID: 3PSC, chains A, B and G). Chain B directly contacts chain A (beta-adrenergic receptor kinase-1) and chain G (guanine nucleotide binding protein gamma subunit). To determine to what extent the interface residues on chain B, which correspond to partner chains A and G, can be distinguished, we predicted interacting residue pairs for the BA and BG complexes and converted them to single-residue predictions for chain B. Figure 6 shows the results of these predictions. Although a number of common true and false positive cases were produced, several residues specific to each partner chain were correctly identified. Such a separation would not be possible with conventional sequence-based prediction methods. However, further analysis is needed to benchmark the current method's ability to distinguish partner-specific interfaces more accurately and quantitatively, and work is in progress toward this goal.

### Application as a docking-pose scoring-function

Pair-wise predictions are likely to be of great value in scoring protein-protein complex *decoy poses* in a docking experiment. One of the first challenges in docking experiments is to select a promising candidate from a set of these decoys, which are generated by treating individual proteins as rigid bodies and sampling their hypothetical complexes [53]. The developers of DBD3.0 used this data set to evaluate the docking program ZDOCK [7], and they provided docking decoy poses that were generated for these complexes using a rigid-body procedure, along with their ranks based on ZDOCK scores and the number of hits in the top 2000 poses.

Using this data set of 15 degree rotation poses, we compared the performance of our proposed method with the ZDOCK ranks. To obtain a rank from our procedure, we predicted the pair-wise scores for each complex (using leave-one-out cross-validation, as described above) and computed the AUC for the predicted scores in reference to the contact data obtained from each pose. The AUC was treated as a scoring function, and all the poses were ranked in order of their AUC. The two evaluation measures used by ZDOCK are (1) the number of native-like hits (native/decoy RMSD<2.5 Å) in the top 2000 poses and (2) the rank of the first native like structure. We found that the number of native-like hits that were obtained using the AUC of our proposed method was approximately 82% of the number of hits produced by ZDOCK. Although we used a straightforward sequence-based approach (e.g., with no repulsive term), this result appears promising and can form the basis of a more elaborate scoring function that would account for structure and other aspects.

We also tested whether our method provided information that was not already available in the ZDOCK scoring procedure. For this, we re-ranked all the complexes by taking a weighted average of the ranks from the ZDOCK and AUC-based scores (the ZDOCK rank was assigned a 3∶1 weight after integer values between 1 and 4 were arbitrarily tested, and the value that produced slightly better results than others was selected). We found a modest overall increase of 3% (from 479 to 493 in the 78 complexes with at least one native-like pose) in the number of hits using this consensus-based approach. However, the rank of the first hit was not improved with either our AUC-based method or a consensus-based approach, which suggests that the AUC-based information is of rather low resolution and cannot be used to rank closely related promising candidates. This problem has been found to be the most difficult to solve by even the best scoring functions that have been developed thus far [53]. Therefore, although agreement between contacts derived from a docking pose and our pair-wise prediction can provide useful added value to the scoring procedures, further work must be performed to take maximum advantage of this observation.

### PPIPP Web server

A two-stage prediction model that was trained on interacting residue pairs, as described above, has been developed and is publicly available at http://tardis.nibio.go.jp/netasa/ppipp/. This online version of our model uses two FASTA formatted sequences as inputs and performs pair-wise predictions between their residues. Final scores are provided for residue pairs, and the scores are also converted to single residues for each chain. A simple graphical representation of the top 200 pairs is also displayed that shows the possible connectivity in the two chains. Further improvements with respect to the graphical presentation of the results are in progress.

### Conclusions

The role of partner information in predicting protein-protein interaction sites has been found to be important; as a result, pair-wise models outperform partner-unaware models. Prediction of the single-protein interface residues that correspond to different partner proteins makes it possible to predict multiple interfaces on the same proteins; it also allows us to accurately pair interacting residues from individual protein chains.

## Supporting Information

Figure S1 **ROC curves for predicting interacting residue pairs from models trained on single sequences (SS) and protein pairs (PP).**
(PDF)

Figure S2 **ROC curves for predicting interacting single residues from models trained on single sequences (SS) and protein pairs (PP).**
(PDF)

Table S1 **Contact preferences of residue pairs (sorted by p-values).** Total number of residue pairs in the entire data set is 7,750,982 of which 11,259 have at least one contact within (one residue of the pair is less than 6.0 Å from the other).
(PDF)

Table S2 **Interacting residue pair prediction performance for each protein-protein complex and characterization of each complex into enzyme, antibody, conformational change (RMSD) and interface size (ΔASA).**
(PDF)
