# Supplementary material for: Partner-Aware Prediction of Interacting Residues in Protein-Protein Complexes from Sequence Data
Source: PLoS One. 2011 Dec 14;6(12):e29104. doi: 10.1371/journal.pone.0029104 (PMC3237601; doi:10.1371/journal.pone.0029104)
Supplement: Figure S1 — ROC curves for predicting interacting residue pairs from models trained on single sequences (SS) and protein pairs (PP). (PDF) [file pone.0029104.s001.pdf]

Figure S1. ROC curves for predicting interacting residue pairs from models trained on single sequences (SS) and protein pairs (PP).

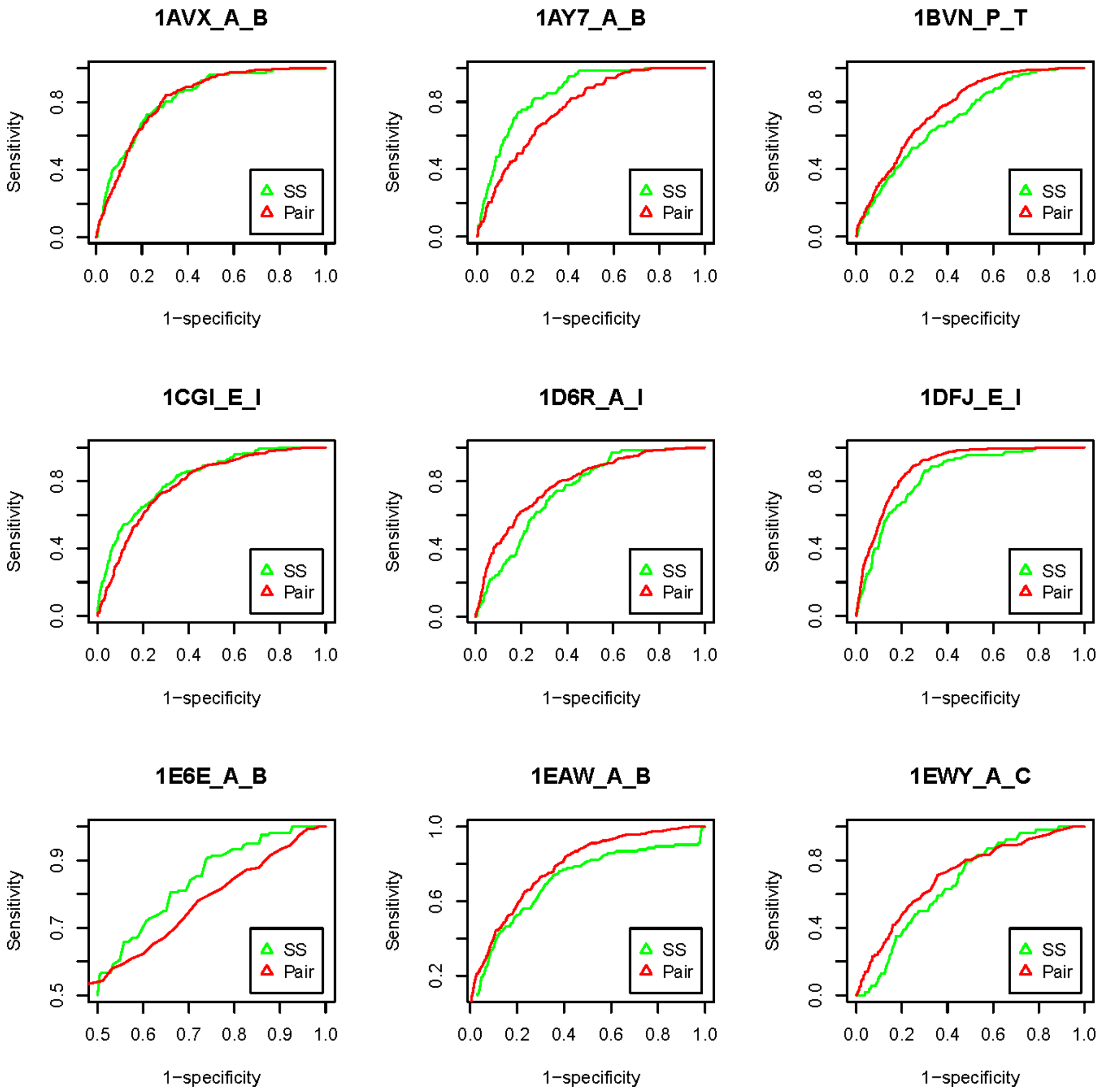

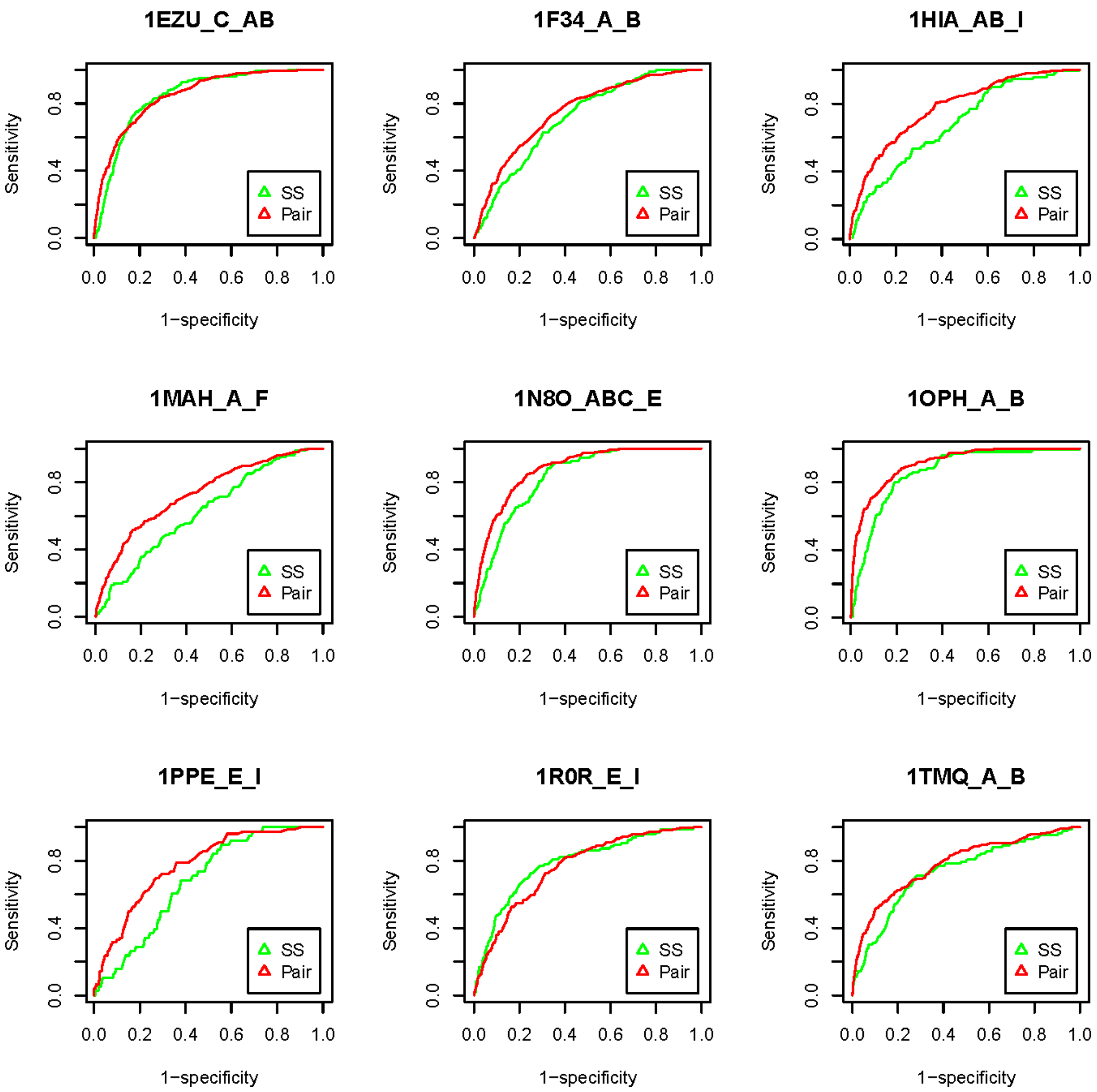
1EZU_C_AB
1F34_A_B
1HIA_AB_I
1MAH_A_F
1N8O_ABC_E
1OPH_A_B
1PPE_E_I
1R0R_E_I
1TMQ_A_B
Sensitivity
1−specificity
0.0 0.4 0.8
0.0 0.2 0.4 0.6 0.8 1.0
SS
Pair

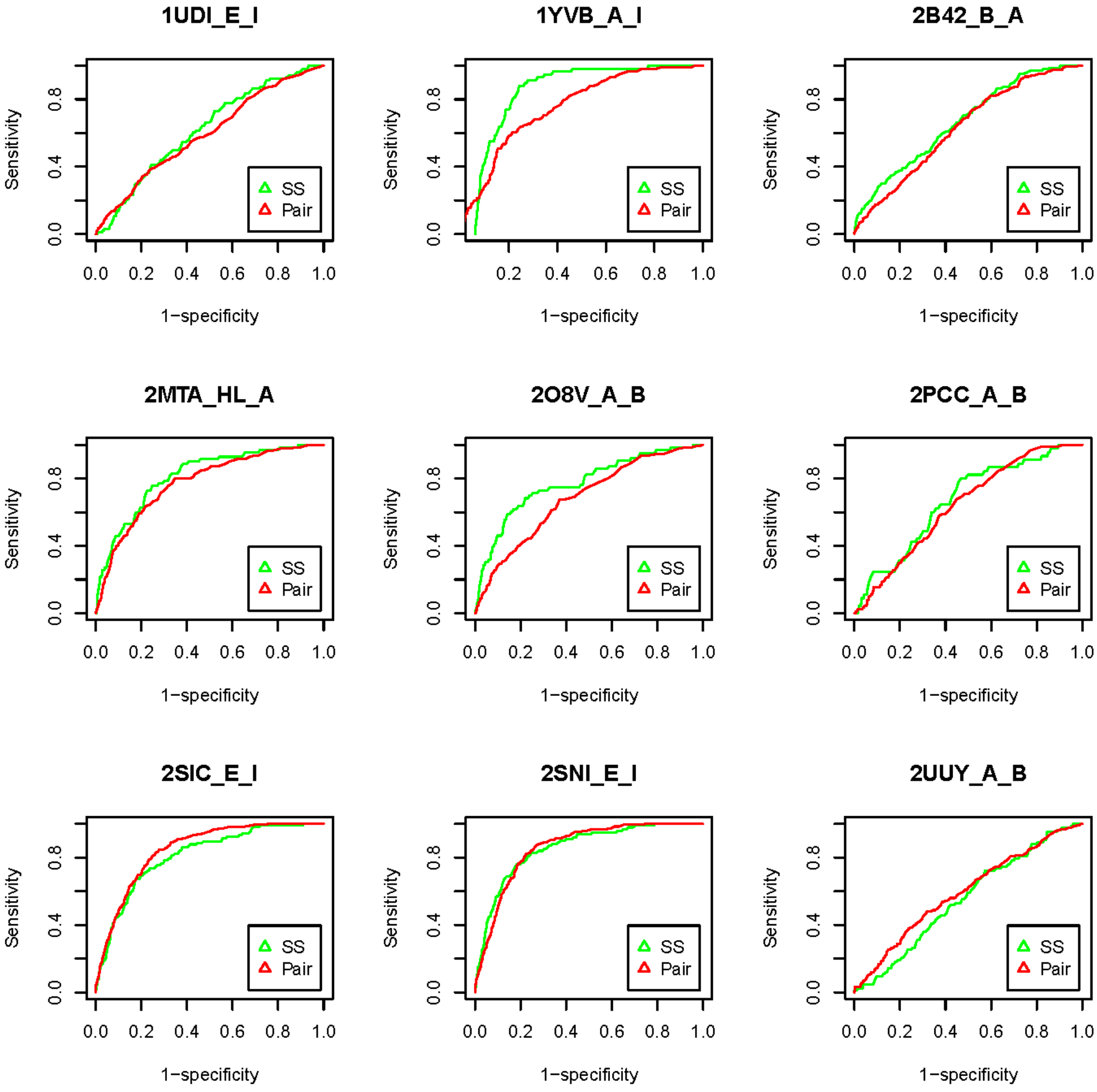

1UDI_E_I
1YVB_A_I
2B42_B_A
2MTA_HL_A
2O8V_A_B
2PCC_A_B
2SIC_E_I
2SNI_E_I
2UUY_A_B
Sensitivity
1−specificity
SS
Pair
0.0
0.2
0.4
0.6
0.8
1.0

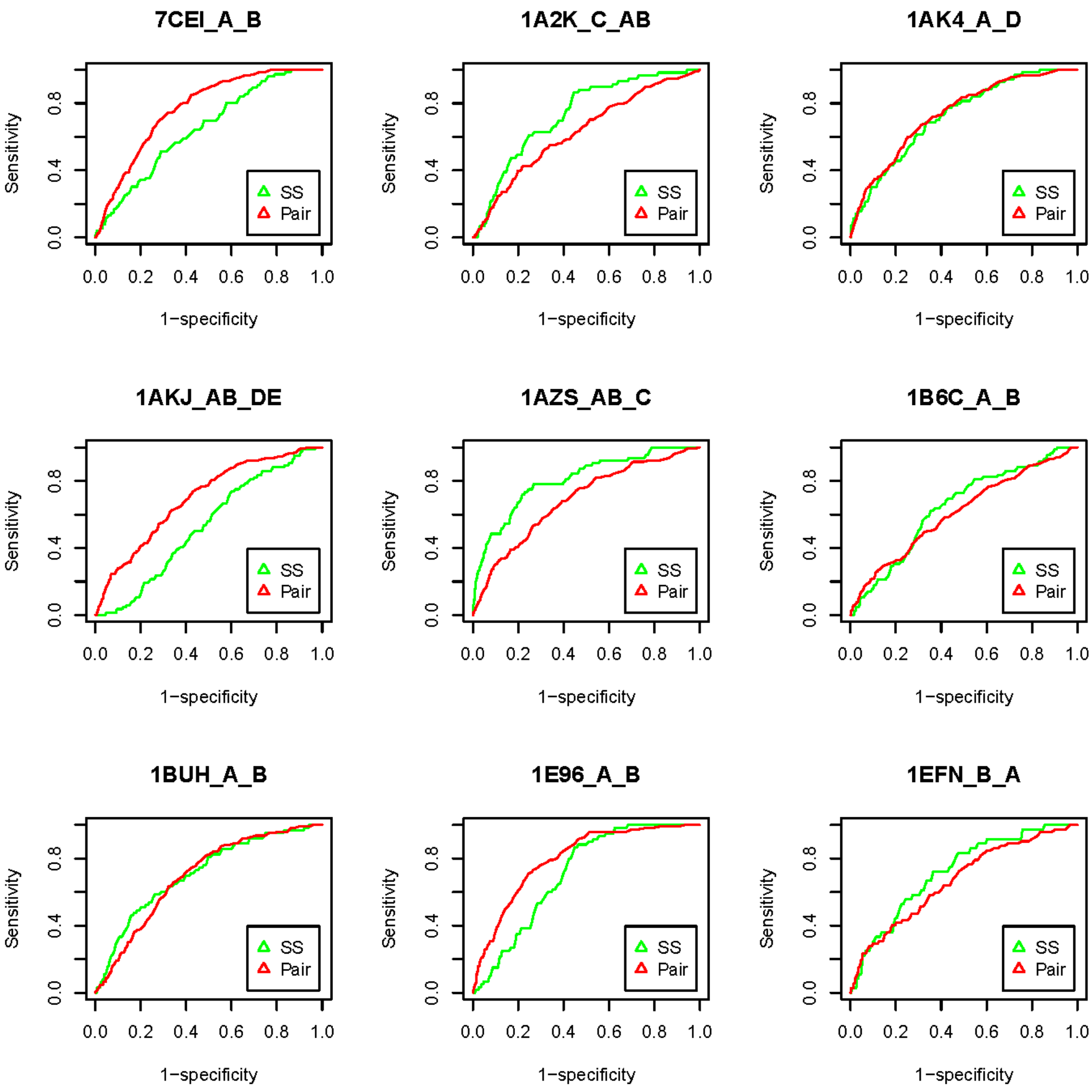
7CEI_A_B
1A2K_C_AB
1AK4_A_D
1AKJ_AB_DE
1AZS_AB_C
1B6C_A_B
1BUH_A_B
1E96_A_B
1EFN_B_A
Sensitivity
0.0
0.4
0.8
0.0 0.2 0.4 0.6 0.8 1.0
1−specificity
SS
Pair

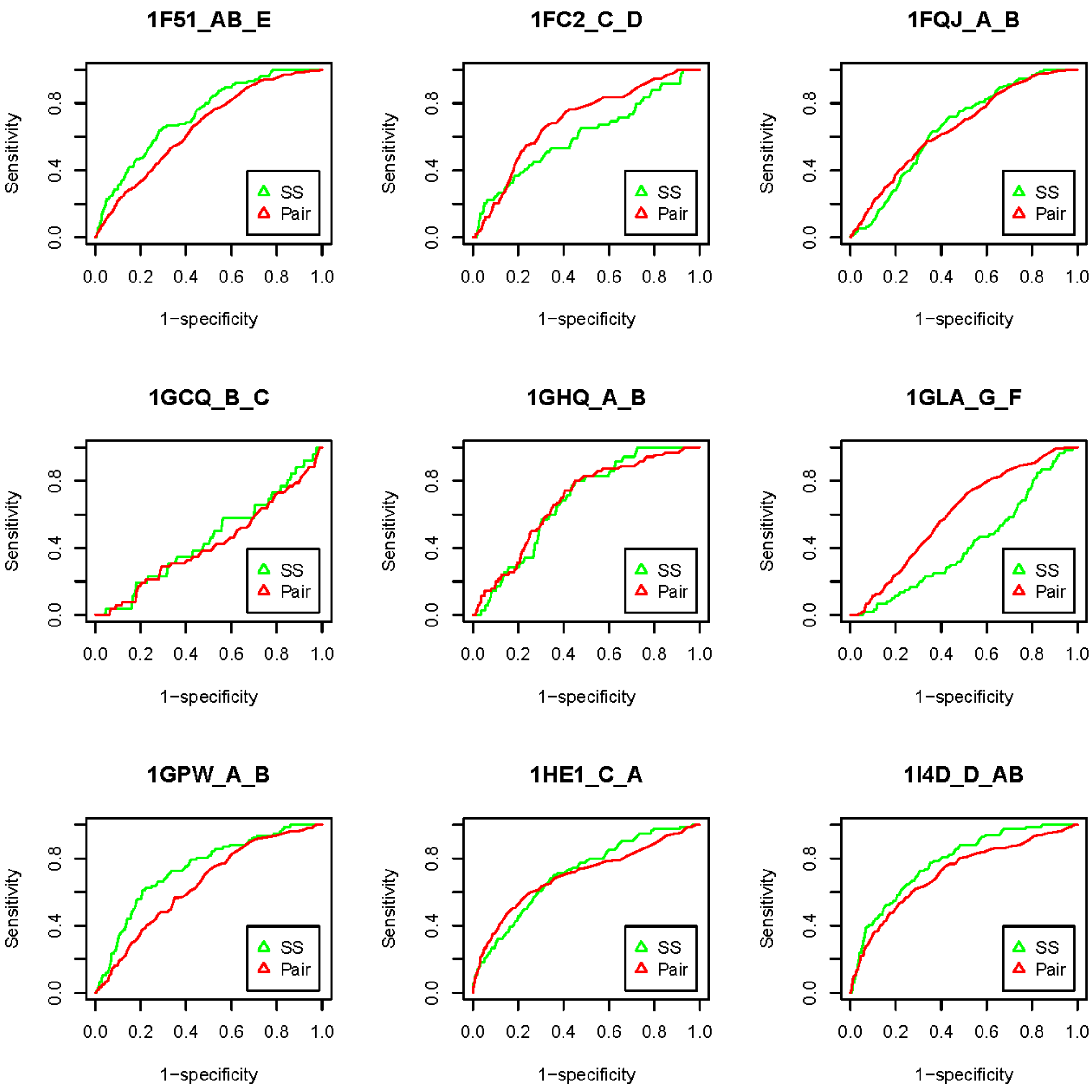
1F51_AB_E
1FC2_C_D
1FQJ_A_B
1GCQ_B_C
1GHQ_A_B
1GLA_G_F
1GPW_A_B
1HE1_C_A
1I4D_D_AB
Sensitivity
1−specificity
0.0
0.2
0.4
0.6
0.8
1.0
SS
Pair

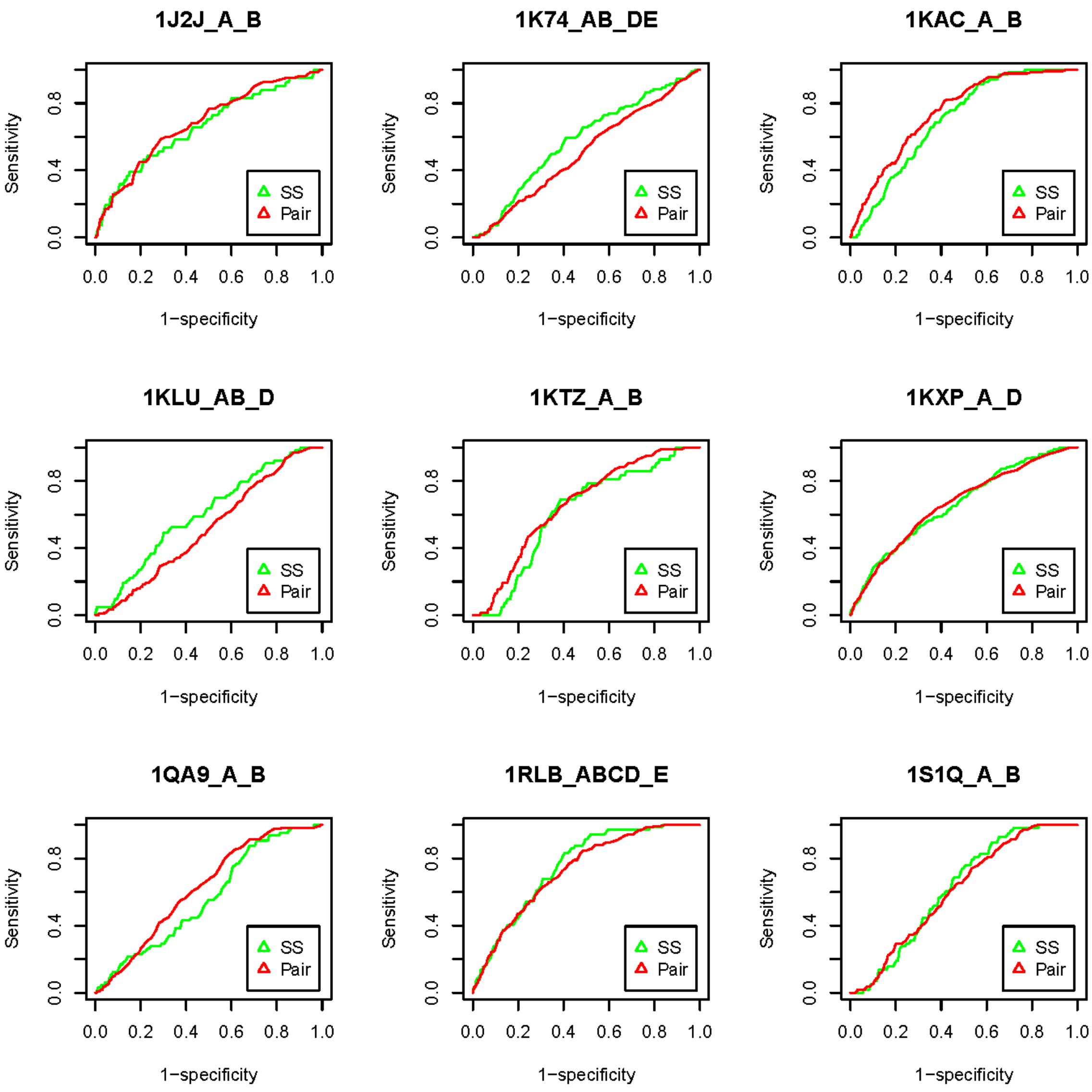

1J2J_A_B
1K74_AB_DE
1KAC_A_B
1KLU_AB_D
1KTZ_A_B
1KXP_A_D
1QA9_A_B
1RLB_ABCD_E
1S1Q_A_B
Sensitivity
1−specificity
0.0
0.4
0.8
0.0
0.2
0.4
0.6
0.8
1.0
SS
Pair

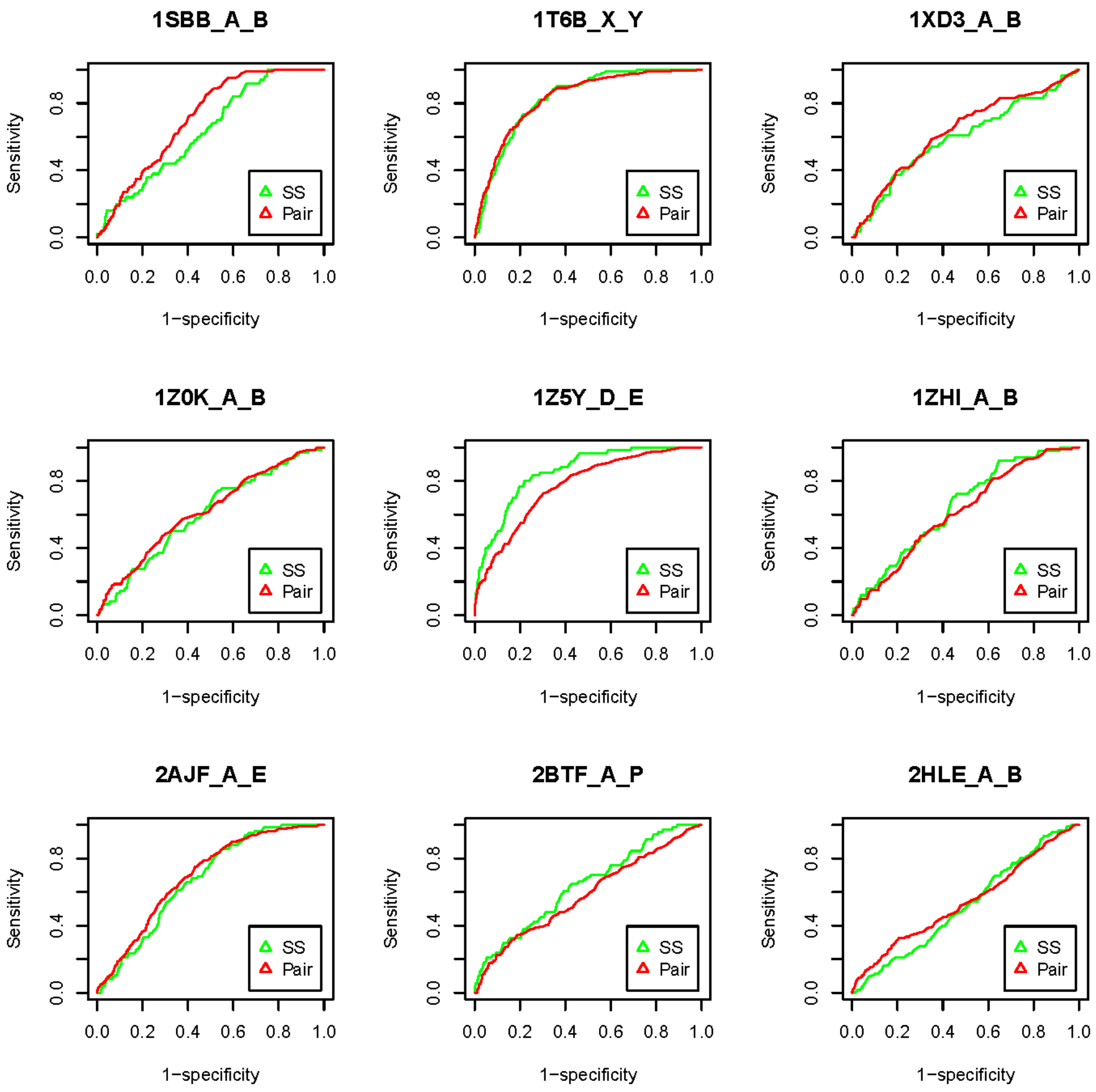

1SBB_A_B
1T6B_X_Y
1XD3_A_B
1Z0K_A_B
1Z5Y_D_E
1ZHI_A_B
2AJF_A_E
2BTF_A_P
2HLE_A_B
Sensitivity
1−specificity
0.0
0.2
0.4
0.6
0.8
1.0
SS
Pair

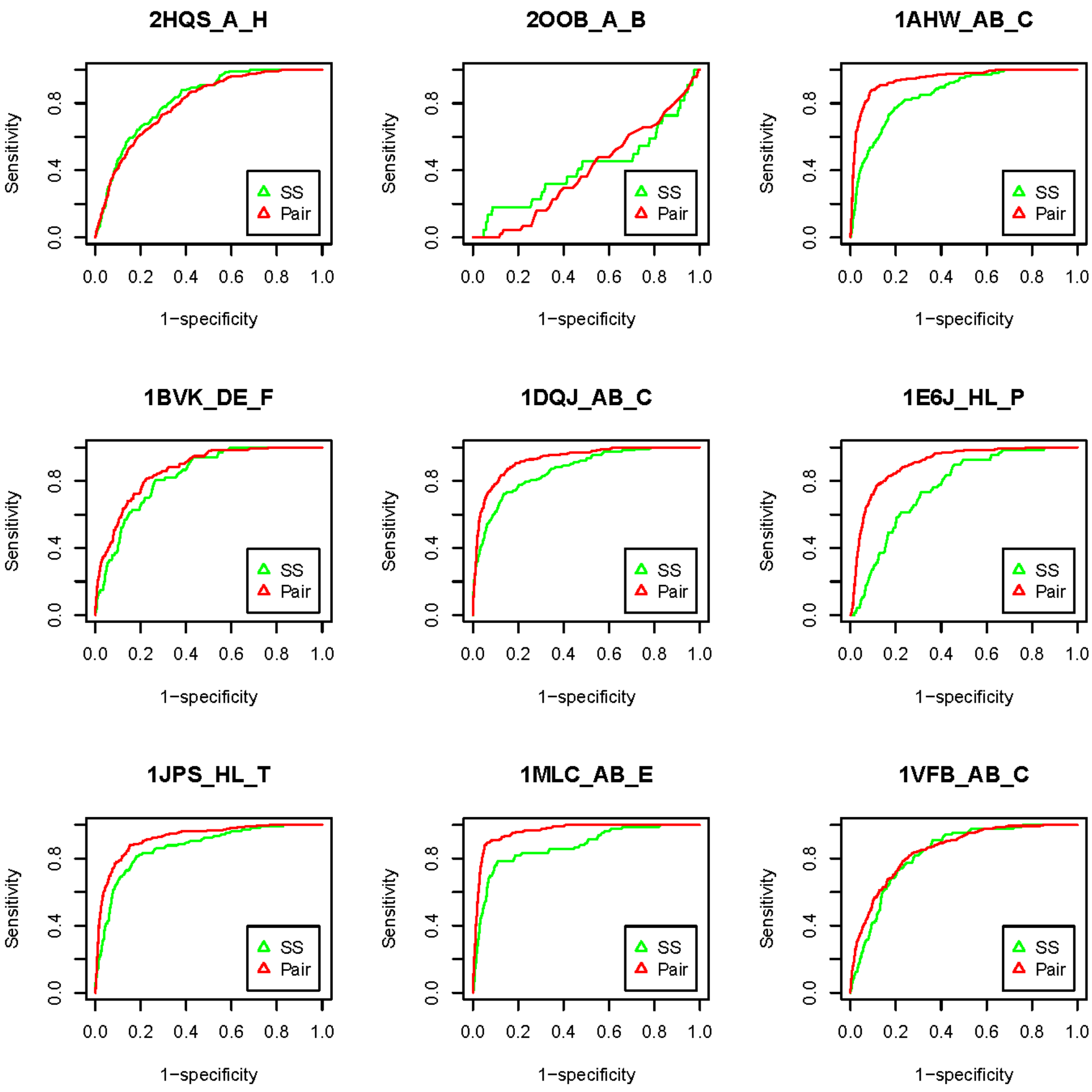
2HQS_A_H
2OOB_A_B
1AHW_AB_C
1BVK_DE_F
1DQJ_AB_C
1E6J_HL_P
1JPS_HL_T
1MLC_AB_E
1VFB_AB_C
Sensitivity
1−specificity
0.0
0.2
0.4
0.6
0.8
1.0
SS
Pair

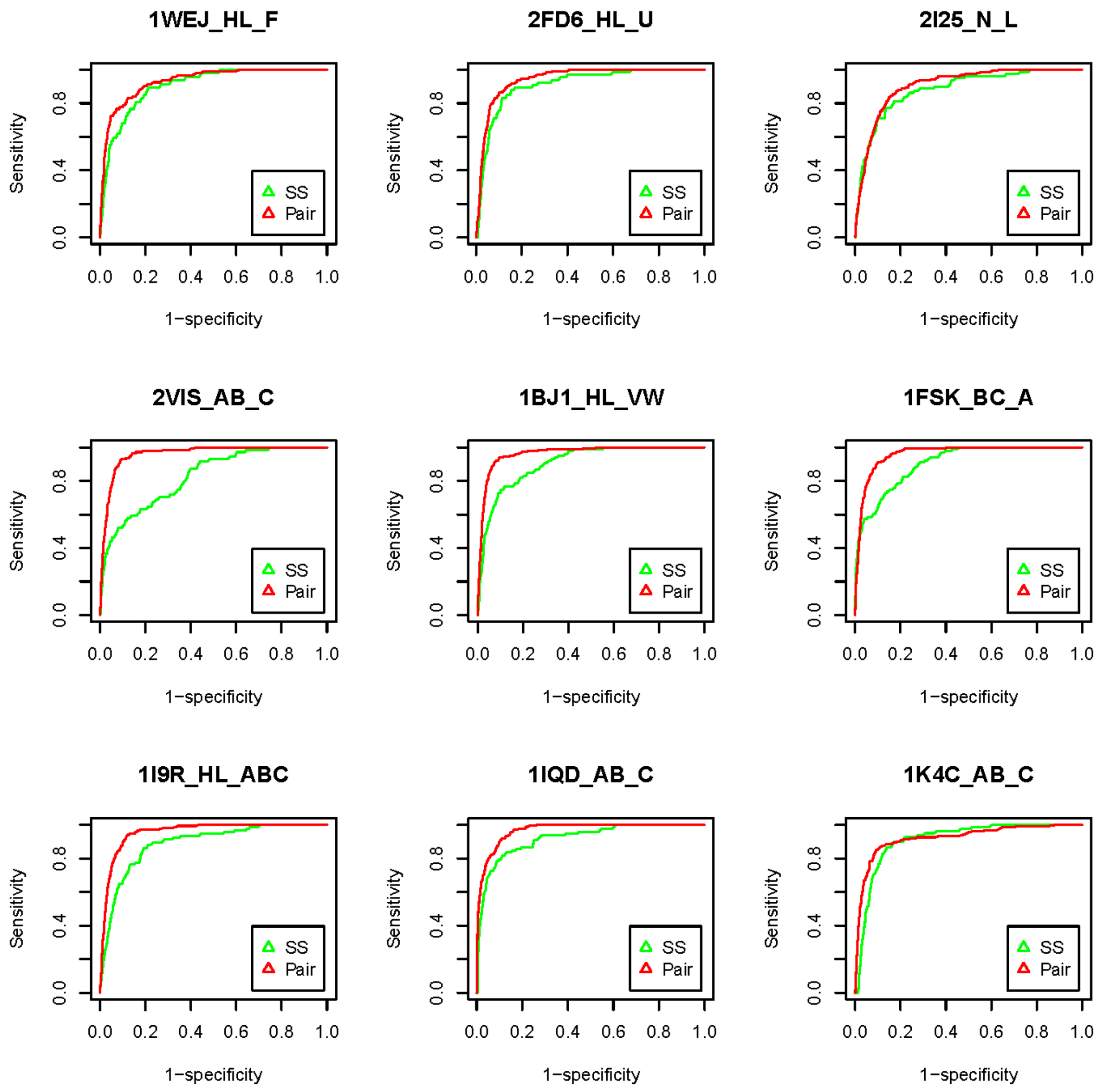
1WEJ_HL_F
2FD6_HL_U
2I25_N_L
2VIS_AB_C
1BJ1_HL_VW
1FSK_BC_A
1I9R_HL_ABC
1IQD_AB_C
1K4C_AB_C
Sensitivity
1−specificity
SS
Pair
0.0
0.2
0.4
0.6
0.8
1.0

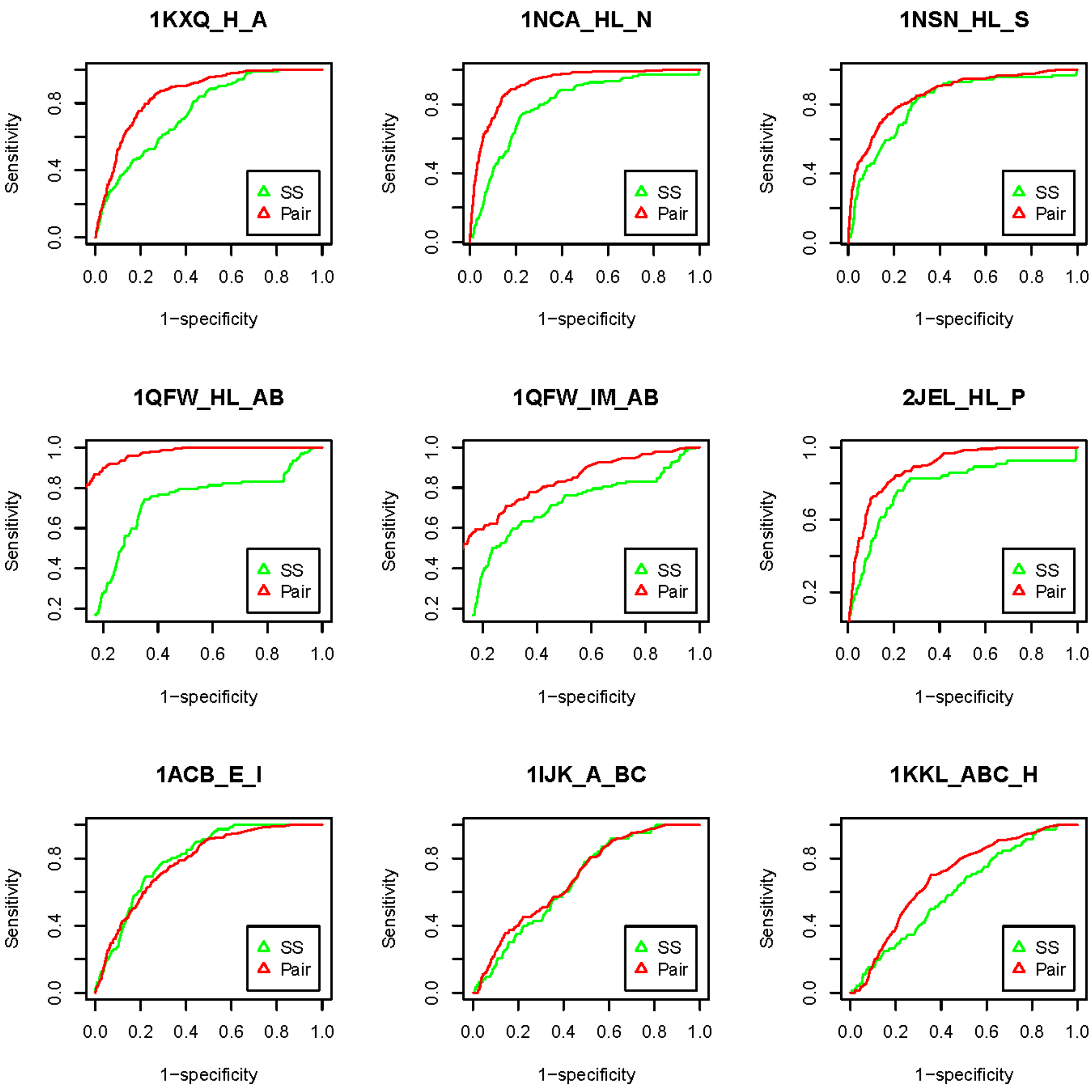
1KXQ_H_A
1NCA_HL_N
1NSN_HL_S
1QFW_HL_AB
1QFW_IM_AB
2JEL_HL_P
1ACB_E_I
1IJK_A_BC
1KKL_ABC_H
Sensitivity
1−specificity
SS
Pair

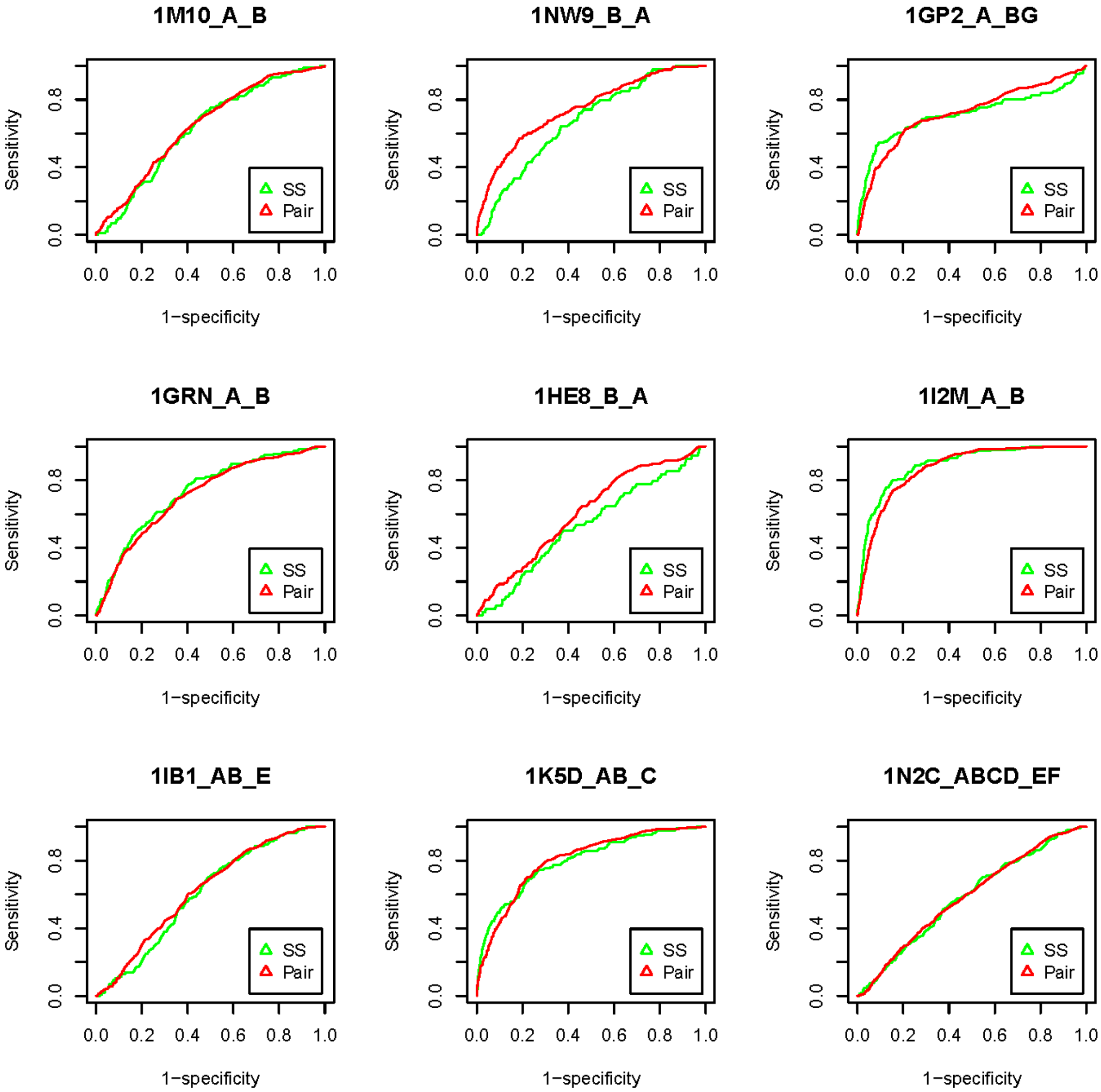

1M10_A_B
1NW9_B_A
1GP2_A_BG
1GRN_A_B
1HE8_B_A
1I2M_A_B
1IB1_AB_E
1K5D_AB_C
1N2C_ABCD_EF
Sensitivity
1−specificity
0.0
0.2
0.4
0.6
0.8
1.0
SS
Pair

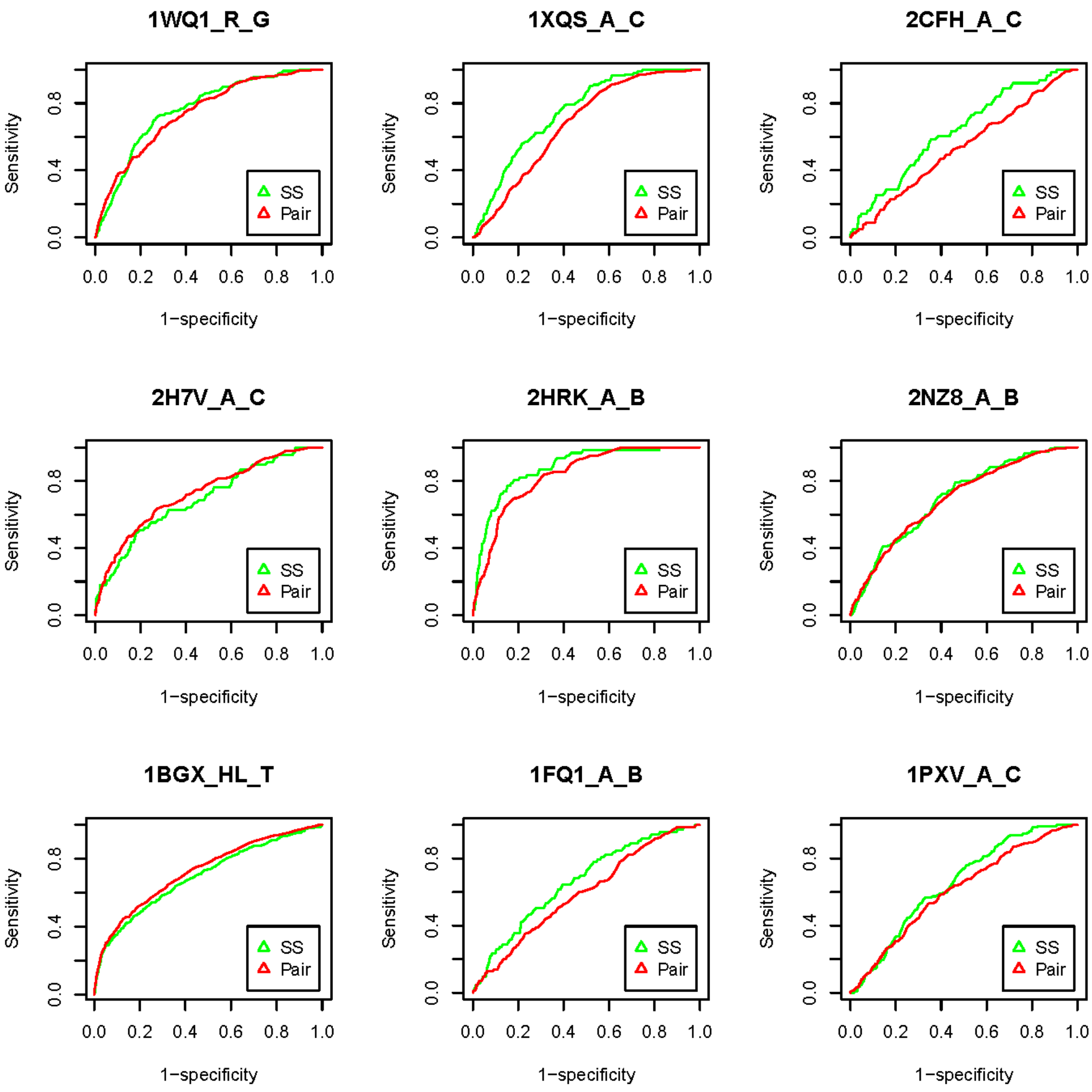
1WQ1_R_G
1XQS_A_C
2CFH_A_C
2H7V_A_C
2HRK_A_B
2NZ8_A_B
1BGX_HL_T
1FQ1_A_B
1PXV_A_C
Sensitivity
0.0
0.4
0.8
1−specificity
0.0
0.2
0.4
0.6
0.8
1.0
SS
Pair

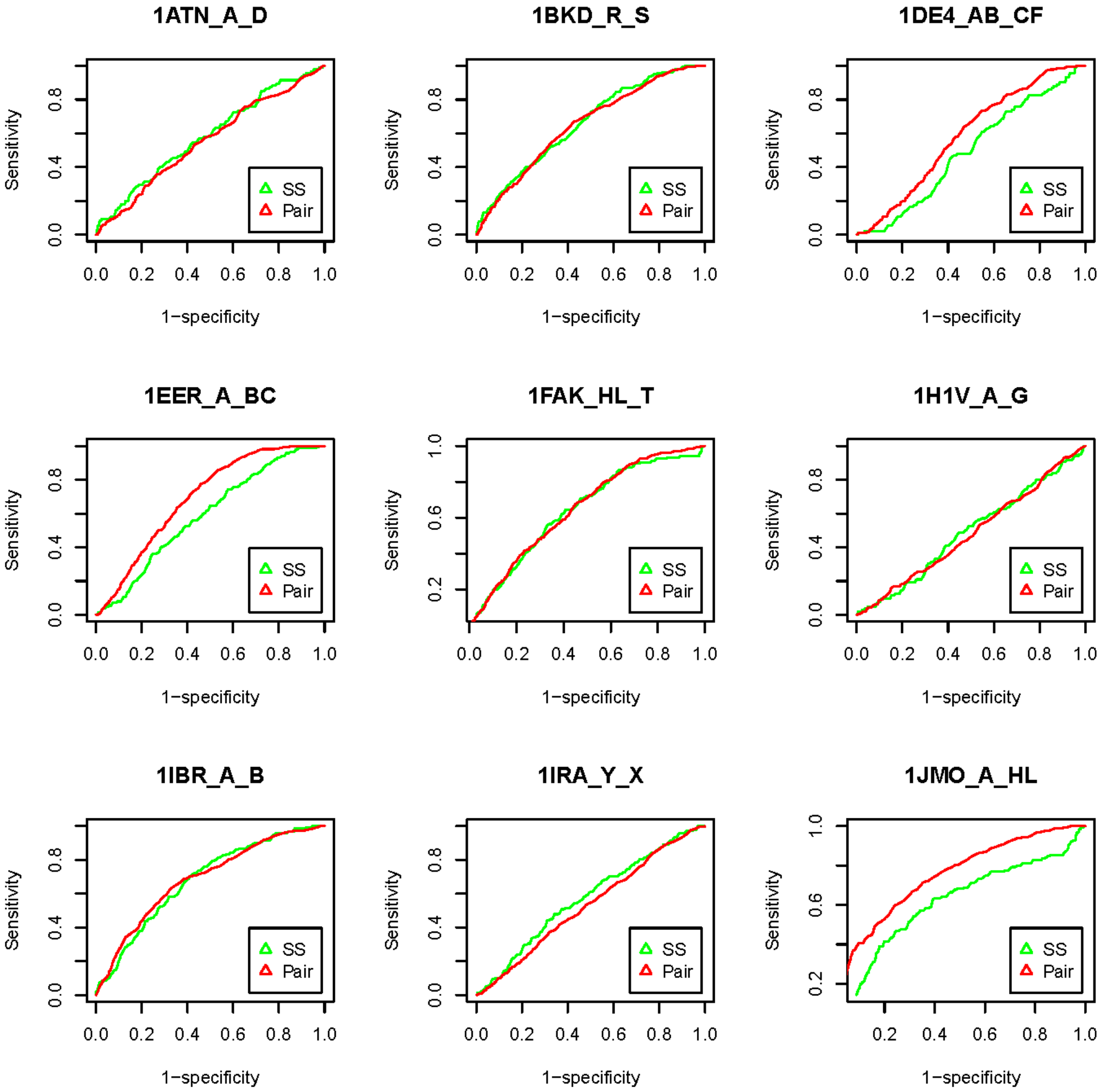
1ATN_A_D
1BKD_R_S
1DE4_AB_CF
1EER_A_BC
1FAK_HL_T
1H1V_A_G
1IBR_A_B
1IRA_Y_X
1JMO_A_HL
Sensitivity
1−specificity
SS
Pair

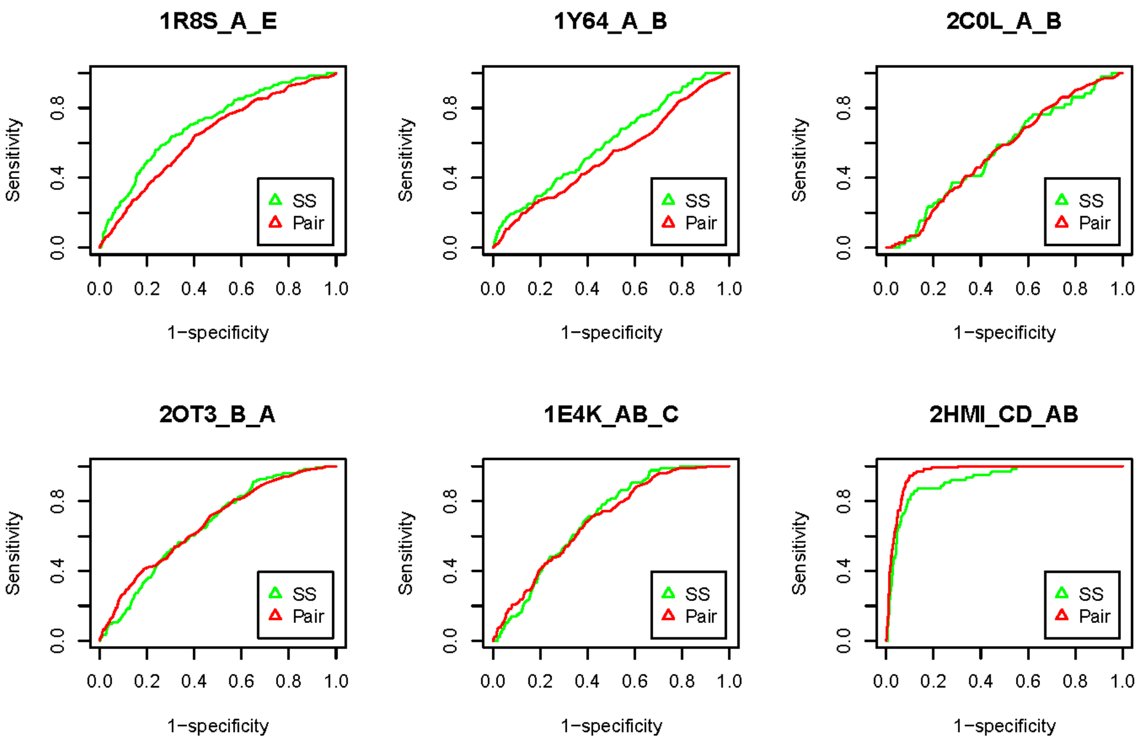
1R8S_A_E
1Y64_A_B
2C0L_A_B
2OT3_B_A
1E4K_AB_C
2HMI_CD_AB
Sensitivity
1−specificity
0.0
0.4
0.8
0.0
0.2
0.4
0.6
0.8
1.0
SS
Pair
