# Supplementary material for: Partner-Aware Prediction of Interacting Residues in Protein-Protein Complexes from Sequence Data
Source: PLoS One. 2011 Dec 14;6(12):e29104. doi: 10.1371/journal.pone.0029104 (PMC3237601; doi:10.1371/journal.pone.0029104)
Supplement: Table S1 — Contact preferences of residue pairs (sorted by p-values). Total number of residue pairs in the entire data set is 7,750,982 of which 11,259 have at least one contact within (one residue of the pair is less than 6.0 Å from the other). (PDF) [file pone.0029104.s003.pdf]

Supplementary data:

Table S1: Contact preferences of residue pairs (sorted by p-values). Total number of residue pairs in the entire data set is 7,750,982 of which 11,259 have at least one contact within (one residue of the pair is less than 6.0Å from the other).

Residue Pair	# Occurrences	Propensity	Observed no. of contacting pairs	Expected no. of contacting pairs	Chi-square	p-value
D-R	38053	2.9	161	55.3	202.2	6.85E-46
R-Y	27214	3.0	119	39.5	159.8	1.28E-36
N-Y	27275	3.0	118	39.6	155.1	1.36E-35
E-R	43020	2.4	152	62.5	128.2	1.01E-29
R-W	10880	3.6	57	15.8	107.4	3.67E-25
N-R	29478	2.4	104	42.8	87.4	8.80E-21
K-Y	38155	2.2	124	55.4	84.9	3.22E-20
D-K	53701	2.0	158	78.0	82.0	1.34E-19
E-K	61956	1.9	172	90.0	74.7	5.42E-18
W-Y	10501	3.2	49	15.3	74.7	5.60E-18
T-Y	40180	2.1	124	58.4	73.8	8.60E-18
Q-Y	24241	2.4	85	35.2	70.4	4.85E-17
D-Y	33431	2.1	101	48.6	56.6	5.27E-14
F-Y	23691	2.3	78	34.4	55.2	1.09E-13
Q-R	26173	2.2	83	38.0	53.2	2.98E-13
A-V	74917	0.3	34	108.8	51.4	7.36E-13
P-Y	29237	2.0	85	42.5	42.6	6.74E-11

M-Y	12988	2.5	47	18.9	42.0	9.35E-11
G-Y	43696	1.8	115	63.5	41.8	9.95E-11
N-Q	26939	2.0	79	39.1	40.6	1.85E-10
A-L	90550	0.5	60	131.5	38.9	4.46E-10
L-V	94746	0.5	65	137.6	38.3	5.99E-10
K-V	69074	0.4	40	100.3	36.3	1.71E-09
H-R	13625	2.3	46	19.8	34.7	3.83E-09
H-Y	12473	2.4	43	18.1	34.2	5.05E-09
E-L	84917	0.5	59	123.3	33.6	6.87E-09
E-V	70674	0.4	45	102.7	32.4	1.26E-08
I-V	57344	0.4	32	83.3	31.6	1.90E-08
A-P	49550	0.3	25	72.0	30.7	3.08E-08
P-W	12271	2.3	41	17.8	30.1	4.04E-08
D-L	74253	0.5	51	107.9	30.0	4.38E-08
S-Y	45371	1.7	110	65.9	29.5	5.59E-08
S-V	81876	0.5	61	118.9	28.2	1.08E-07
A-E	67785	0.5	46	98.5	28.0	1.24E-07
A-D	58846	0.5	39	85.5	25.3	4.98E-07
N-T	44581	1.6	105	64.8	25.0	5.71E-07
P-V	50955	0.4	31	74.0	25.0	5.73E-07
A-K	66873	0.5	49	97.1	23.9	1.04E-06
Q-W	9977	2.3	33	14.5	23.6	1.16E-06
A-S	78135	0.5	62	113.5	23.4	1.34E-06

L-S	99102	0.6	86	144.0	23.3	1.36E-06
A-A	35784	0.3	18	52.0	22.2	2.44E-06
D-N	37447	1.6	89	54.4	22.0	2.71E-06
I-T	51263	0.5	34	74.5	22.0	2.74E-06
T-V	71896	0.5	57	104.4	21.5	3.46E-06
R-R	14846	2.0	43	21.6	21.3	3.92E-06
H-W	4767	2.7	19	6.9	21.1	4.46E-06
C-T	21962	0.2	6	31.9	21.0	4.52E-06
A-I	55011	0.5	40	79.9	19.9	8.03E-06
G-W	18160	1.9	49	26.4	19.4	1.06E-05
E-Y	39234	1.6	90	57.0	19.1	1.23E-05
A-G	75458	0.6	64	109.6	19.0	1.32E-05
K-L	84632	0.6	75	122.9	18.7	1.54E-05
R-T	44625	1.5	99	64.8	18.0	2.18E-05
L-Y	53527	1.5	115	77.8	17.8	2.40E-05
T-W	17879	1.8	47	26.0	17.0	3.68E-05
E-I	51844	0.5	40	75.3	16.6	4.73E-05
I-Y	30846	1.6	72	44.8	16.5	4.86E-05
D-Q	33025	1.6	76	48.0	16.4	5.19E-05
G-L	92001	0.7	88	133.6	15.6	7.88E-05
G-V	79029	0.6	74	114.8	14.5	1.40E-04
L-T	87113	0.7	84	126.5	14.3	1.56E-04
D-V	61006	0.6	54	88.6	13.5	2.36E-04

E-N	42843	1.5	91	62.2	13.3	2.66E-04
V-V	39454	0.5	30	57.3	13.0	3.09E-04
L-P	61194	0.6	55	88.9	12.9	3.25E-04
N-N	14801	1.8	38	21.5	12.7	3.73E-04
N-W	11394	1.9	31	16.6	12.6	3.83E-04
A-T	69472	0.7	66	100.9	12.1	5.10E-04
F-V	42113	0.6	34	61.2	12.1	5.12E-04
C-E	20611	0.4	11	29.9	12.0	5.38E-04
G-P	51288	0.6	45	74.5	11.7	6.31E-04
G-R	48265	1.4	98	70.1	11.1	8.65E-04
R-S	51783	1.4	104	75.2	11.0	9.05E-04
A-C	22512	0.4	14	32.7	10.7	1.07E-03
W-W	2238	2.8	9	3.3	10.2	1.43E-03
F-R	26363	1.5	58	38.3	10.1	1.45E-03
H-H	3036	2.5	11	4.4	9.8	1.70E-03
A-F	40595	0.6	35	59.0	9.7	1.80E-03
C-V	23556	0.5	16	34.2	9.7	1.84E-03
I-P	36174	0.6	30	52.5	9.7	1.87E-03
G-S	80844	0.7	84	117.4	9.5	2.03E-03
H-N	13952	1.7	34	20.3	9.3	2.28E-03
A-Q	40920	0.6	37	59.4	8.5	3.61E-03
D-W	13491	1.6	32	19.6	7.9	5.08E-03
K-W	15686	1.6	36	22.8	7.7	5.63E-03

F-M	13046	1.6	31	19.0	7.7	5.64E-03
C-D	17876	0.5	12	26.0	7.5	6.13E-03
P-R	30234	1.4	62	43.9	7.4	6.36E-03
D-I	45422	0.7	44	66.0	7.3	6.81E-03
Q-T	40265	1.4	79	58.5	7.2	7.32E-03
C-N	14703	0.4	9	21.4	7.2	7.50E-03
G-Q	42084	1.3	82	61.1	7.1	7.60E-03
I-S	57812	0.7	60	84.0	6.8	8.88E-03
E-G	69737	0.7	75	101.3	6.8	8.97E-03
E-H	19960	1.5	43	29.0	6.8	9.29E-03
I-K	50360	0.7	51	73.2	6.7	9.60E-03
C-P	15286	0.5	10	22.2	6.7	9.60E-03
K-K	29365	0.6	26	42.7	6.5	1.08E-02
A-M	23170	0.6	19	33.7	6.4	1.15E-02
L-N	57843	0.7	61	84.0	6.3	1.20E-02
Y-Y	12047	1.6	28	17.5	6.3	1.21E-02
K-M	21159	0.6	17	30.7	6.1	1.32E-02
C-M	6982	1.8	18	10.1	6.1	1.36E-02
D-P	38923	0.7	38	56.5	6.1	1.37E-02
S-W	20452	1.4	43	29.7	5.9	1.47E-02
G-N	49192	1.3	92	71.5	5.9	1.51E-02
H-I	16090	1.5	35	23.4	5.8	1.62E-02
C-R	14480	1.5	32	21.0	5.7	1.68E-02

H-M	6686	1.8	17	9.7	5.5	1.94E-02
N-S	50999	1.3	94	74.1	5.4	2.07E-02
N-V	48750	0.7	52	70.8	5.0	2.54E-02
M-V	24024	0.6	22	34.9	4.8	2.90E-02
M-S	23103	1.4	46	33.6	4.6	3.17E-02
M-W	4987	1.8	13	7.2	4.6	3.25E-02
Q-V	43304	0.7	46	62.9	4.5	3.31E-02
P-T	48789	0.7	53	70.9	4.5	3.38E-02
P-S	55822	0.8	62	81.1	4.5	3.40E-02
L-W	21473	1.4	43	31.2	4.5	3.45E-02
F-Q	23442	1.4	46	34.1	4.2	4.06E-02
E-W	15520	1.4	32	22.5	4.0	4.64E-02
F-G	41615	0.7	45	60.4	3.9	4.69E-02
V-Y	43763	1.2	79	63.6	3.7	5.30E-02
I-W	12404	1.4	26	18.0	3.5	6.00E-02
A-N	45622	0.8	51	66.3	3.5	6.07E-02
E-Q	37539	1.2	68	54.5	3.3	6.81E-02
L-M	28451	1.3	53	41.3	3.3	6.94E-02
M-R	14975	1.4	30	21.8	3.1	7.70E-02
F-S	42429	0.8	48	61.6	3.0	8.25E-02
I-M	18139	0.7	18	26.3	2.6	1.04E-01
D-H	17749	1.3	34	25.8	2.6	1.06E-01
G-I	56920	0.8	68	82.7	2.6	1.06E-01

K-T	63588	0.8	77	92.4	2.6	1.10E-01
K-N	42560	1.2	74	61.8	2.4	1.21E-01
A-H	21795	0.7	23	31.7	2.4	1.24E-01
D-E	55562	0.8	67	80.7	2.3	1.27E-01
E-E	31135	0.8	35	45.2	2.3	1.28E-01
F-H	12163	1.4	24	17.7	2.3	1.32E-01
C-C	3803	0.4	2	5.5	2.2	1.34E-01
N-P	32199	1.2	57	46.8	2.2	1.35E-01
C-K	20752	0.7	22	30.1	2.2	1.38E-01
A-Y	42263	1.2	73	61.4	2.2	1.38E-01
K-P	44693	0.8	53	64.9	2.2	1.39E-01
P-Q	28573	1.2	51	41.5	2.2	1.41E-01
H-Q	12310	1.3	24	17.9	2.1	1.48E-01
G-K	69084	0.9	86	100.4	2.1	1.52E-01
Q-S	46310	1.2	79	67.3	2.0	1.53E-01
D-T	54596	1.2	92	79.3	2.0	1.54E-01
I-Q	30732	1.2	54	44.6	2.0	1.61E-01
H-V	22703	0.8	25	33.0	1.9	1.65E-01
L-R	58812	0.9	73	85.4	1.8	1.79E-01
C-L	27841	0.8	32	40.4	1.8	1.84E-01
E-T	64381	1.1	106	93.5	1.7	1.97E-01
H-S	23095	1.2	41	33.5	1.7	1.98E-01
F-F	11000	1.3	21	16.0	1.6	2.09E-01

G-M	24633	1.2	43	35.8	1.5	2.28E-01
H-L	27197	1.2	47	39.5	1.4	2.33E-01
E-M	21759	1.2	38	31.6	1.3	2.55E-01
E-P	44852	0.9	56	65.2	1.3	2.57E-01
F-P	26821	1.2	46	39.0	1.3	2.59E-01
K-S	73486	1.1	118	106.7	1.2	2.76E-01
D-G	60703	0.9	78	88.2	1.2	2.78E-01
P-P	16735	0.8	19	24.3	1.2	2.82E-01
M-N	14303	0.8	16	20.8	1.1	2.95E-01
E-F	38692	1.1	64	56.2	1.1	2.98E-01
D-F	32970	0.9	41	47.9	1.0	3.19E-01
L-L	57814	0.9	75	84.0	1.0	3.27E-01
S-T	76621	0.9	101	111.3	1.0	3.29E-01
C-Q	13200	0.8	15	19.2	0.9	3.40E-01
K-Q	38020	1.1	62	55.2	0.8	3.62E-01
F-T	37566	0.9	48	54.6	0.8	3.74E-01
F-W	9456	1.2	17	13.7	0.8	3.78E-01
F-K	36660	0.9	47	53.3	0.7	3.92E-01
M-Q	12015	1.2	21	17.5	0.7	3.96E-01
D-S	60686	1.1	96	88.2	0.7	4.03E-01
D-M	19320	0.9	24	28.1	0.6	4.43E-01
H-P	14168	1.2	24	20.6	0.6	4.51E-01
I-L	69156	0.9	93	100.5	0.6	4.57E-01

E-S	74564	1.1	116	108.3	0.5	4.60E-01
G-H	21942	1.1	36	31.9	0.5	4.65E-01
I-N	34402	0.9	45	50.0	0.5	4.82E-01
I-I	21194	0.9	27	30.8	0.5	4.95E-01
H-K	19778	1.1	32	28.7	0.4	5.42E-01
C-Y	13420	1.1	22	19.5	0.3	5.70E-01
M-T	20607	1.1	33	29.9	0.3	5.75E-01
F-N	25901	1.1	41	37.6	0.3	5.82E-01
A-W	17697	0.9	23	25.7	0.3	5.93E-01
V-W	18293	0.9	24	26.6	0.2	6.18E-01
M-P	14931	1.1	24	21.7	0.2	6.20E-01
A-R	46877	0.9	64	68.1	0.2	6.20E-01
M-M	4001	1.2	7	5.8	0.2	6.22E-01
H-T	20195	1.1	32	29.3	0.2	6.23E-01
Q-Q	11791	1.1	19	17.1	0.2	6.51E-01
F-L	51234	1.0	71	74.4	0.2	6.92E-01
K-R	42060	1.0	64	61.1	0.1	7.10E-01
C-F	12793	0.9	17	18.6	0.1	7.13E-01
C-G	24164	0.9	33	35.1	0.1	7.23E-01
I-R	34720	1.0	48	50.4	0.1	7.32E-01
G-G	40228	1.0	61	58.4	0.1	7.37E-01
T-T	33977	1.0	47	49.4	0.1	7.37E-01
G-T	72141	1.0	108	104.8	0.1	7.54E-01

S-S	41914	1.0	63	60.9	0.1	7.86E-01
C-W	5667	1.1	9	8.2	0.1	7.89E-01
R-V	48201	1.0	68	70.0	0.1	8.10E-01
L-Q	52287	1.0	74	76.0	0.1	8.23E-01
F-I	30741	1.0	46	44.7	0.0	8.40E-01
C-S	25630	1.0	38	37.2	0.0	9.00E-01
C-H	6645	1.0	10	9.7	0.0	9.11E-01
D-D	23855	1.0	34	34.7	0.0	9.12E-01
C-I	16712	1.0	24	24.3	0.0	9.55E-01
