# Supplementary material for: Partner-Aware Prediction of Interacting Residues in Protein-Protein Complexes from Sequence Data
Source: PLoS One. 2011 Dec 14;6(12):e29104. doi: 10.1371/journal.pone.0029104 (PMC3237601; doi:10.1371/journal.pone.0029104)
Supplement: Table S2 — Interacting residue pair prediction performance for each protein-protein complex and characterization of each complex into enzyme, antibody, conformational change (RMSD) and interface size (ΔASA). (PDF) [file pone.0029104.s004.pdf]

Table S2. Interacting residue pair prediction performance for each protein-protein complex and characterization of each complex into enzyme, antibody, conformational change (RMSD) and interface size (ΔASA).

Complex name	Difficulty level	Protein-1	Protein-2	RMSD (Å)	ΔASA ($Å^2$)	AUC	Category
1A2K_C_AB	RB	Ran GTPase	Nuclear transport factor 2	1.11	1603	0.63	O
1ACB_E_I	Medium	Chymotrypsin	Eglin C	2.26	1544	0.78	E
1AHW_AB_C	RB	Fab 5g9	Tissue factor	0.69	1899	0.94	A/B
1AK4_A_D	RB	Cyclophilin	HIV capsid	1.33	1029	0.74	O
1AKJ_AB_DE	RB	MHC Class 1 HLA-A2	T-cell CD8 coreceptor	1.14	1995	0.70	O
1ATN_A_D	Difficult	Actin	Dnase I	3.28	1774	0.55	O
1AVX_A_B	RB	Porcine trypsin	Soybean trypsin inhibitor	0.47	1585	0.82	E
1AY7_A_B	RB	Barnase	Barstar	0.54	1237	0.77	E
1AZS_AB_C	RB	Adenylyl cyclase	AC activator Gs alpha complex	0.72	1911	0.69	O
1B6C_A_B	RB	FKBP binding protein	TGFbeta receptor	1.96	1752	0.61	O
1BGX_HL_T	Medium	Fab	Taq polymerase	1.48	5814	0.73	A/B
1BJ1_HL_VW	RB	Fab	vEGF	0.5	1731	0.96	A/B
1BKD_R_S	Difficult	Ras GTPase	Son of Sevenless	2.86	3163	0.65	O
1BUH_A_B	RB	CDK2 kinase	Ckshs1	0.75	1324	0.70	O
1BVK_DE_F	RB	Fv Hulys11	HEW lysozyme	1.24	1321	0.86	A/B
1BVN_P_T	RB	alpha-amylase	Tendamistat	0.87	2222	0.76	E
1CGI_E_I	RB	Bovine chymotrypsinogen	PSTI	2.02	2053	0.78	E
1D6R_A_I	RB	Bovine trypsin	Bowman-Birk inhibitor	1.14	1408	0.79	E
1DE4_AB_CF	Difficult	beta2-microglobulin	Transferrin receptor ectodom.	2.59	2066	0.59	O
1DFJ_E_I	RB	Ribonuclease A	Rnase inhibitor	1.02	2582	0.88	E
1DQJ_AB_C	RB	Fab Hyhel63	HEW lysozyme	0.75	1765	0.93	A/B
1E4K_AB_C	Difficult	FC fragment of human IgG 1	Human FCGR III	2.59	1634	0.69	A/B
1E6E_A_B	RB	Adrenoxin reductase	Adrenoxin	1.33	2315	0.56	E
1E6J_HL_P	RB	Fab	HIV-1 capsid protein p24	1.05	1245	0.90	A/B

1E96_A_B	RB	Rac GTPase	p67 Phox	0.71	1179	0.80	O
1EAW_A_B	RB	Matriptase	BPTI	0.54	1866	0.79	E
1EER_A_BC	Difficult	Erythropoietin	EPO receptor	2.44	3347	0.69	O
1EFN_B_A	RB	HIV-1-NEF protein	SH3 domain	0.77	1254	0.67	O
1EWY_A_C	RB	Ferredoxin reductase	Ferredoxin	0.8	1502	0.71	E
1EZU_C_AB	RB	D102N Trypsin	Ecotin	1.21	2751	0.85	E
1F34_A_B	RB	Porcine pepsin	Ascaris inhibitor 3	0.93	3038	0.75	E
1F51_AB_E	RB	Sporulation response factor B	Sporulation response factor F	0.74	2407	0.66	O
1FAK_HL_T	Difficult	Coagulation factor VIIa	Soluble tissue factor	6.18	3363	0.65	O
1FC2_C_D	RB	Staphylococcu s Protein A	Human Fc fragment	1.69	1307	0.69	O
1FQ1_A_B	Difficult	CDK2 kinase	CDK inhibitor 3	3.41	1832	0.59	E
1FQJ_A_B	RB	Gt-alpha	RGS9	0.91	1806	0.65	O
1FSK_BC_A	RB	Fab	Birch pollen antigen Bet V1	0.45	1623	0.96	A/B
1GCQ_B_C	RB	GRB2 C-ter SH3 domain	GRB2 N-ter SH3 domain	0.92	1208	0.42	O
1GHQ_A_B	RB	Complement C3	Epstein-Barr virus receptor CR2	0.34	800	0.68	O
1GLA_G_F	RB	Glycerol Kinase	Glucose specific phosphocarr ier	0.98	1304	0.60	O
1GP2_A_BG	Medium	Gi-alpha	Gi-beta,gamma	1.65	2287	0.72	O
1GPW_A_B	RB	HISF protein	Amidotransf erase HISH	0.65	2097	0.65	O
1GRN_A_B	Medium	CDC42 GTPase	CDC42 GAP	1.22	2332	0.71	O
1H1V_A_G	Difficult	Actin	Gelsolin	6.62	2071	0.48	O
1HE1_C_A	RB	Rac GTPase	Pseudomon as toxin GAP dom.	0.93	2113	0.70	O
1HE8_B_A	Medium	Ras GTPase	PIP3 kinase	0.92	1305	0.61	O
1HIA_AB_I	RB	Kallikrein	Hirustatin	1.4	1737	0.78	E
1I2M_A_B	Medium	Ran GTPase	RCC1	2.12	2779	0.87	O
1I4D_D_AB	RB	Rac GTPase	Arfaptin	1.41	1657	0.71	O
1I9R_HL_ABC	RB	Fab	Cd40 ligand	1.3	1498	0.96	A/B
1IB1_AB_E	Medium	14-3-3 protein	Serotonin N-acteylase	2.09	2808	0.62	O
1IBR_A_B	Difficult	Ran GTPase	Importin beta	2.54	2270	0.69	O
1IJK_A_BC	Medium	Von Willebrand Factor dom. A1	Botrocetin	0.68	1648	0.68	E

1IQD_AB_C	RB	Fab	Factor VIII domain C2	0.48	1976	0.97	A/B
1IRA_Y_X	Difficult	Interleukin-1 receptor	Interleukin-1 receptor antagonist protein	8.38	3367	0.53	O
1J2J_A_B	RB	Arf1 GTPase	GAT domain of GGA1	0.63	1209	0.68	O
1JMO_A_HL	Difficult	Heparin cofactor	Thrombin	3.21	3461	0.75	O
1JPS_HL_T	RB	Fab D3H44	Tissue factor	0.51	1852	0.92	A
1K4C_AB_C	RB	Fab	Potassium Channel Kcsa	0.53	1601	0.92	A/B
1K5D_AB_C	Medium	Ran GTPase	Ran GAP	1.19	2527	0.80	O
1K74_AB_DE	RB	RXR-alpha	PPAR-gamma	0.8	2200	0.51	O
1KAC_A_B	RB	Adenovirus fiber knob protein	Adenovirus receptor	0.95	1456	0.75	O
1KKL_ABC_H	Medium	HPr kinase C-ter domain	HPr	2.2	1641	0.69	E
1KLU_AB_D	RB	MHC class 2 HLA-DR1	Staphylococcus enterotoxin C3	0.43	1254	0.52	O
1KTZ_A_B	RB	TGF-beta	TGF-beta receptor	0.39	989	0.66	O
1KXP_A_D	RB	Actin	Vitamin D binding protein	1.12	3341	0.66	O
1KXQ_H_A	RB	camel VHH	Pancreatic alpha-amylase	0.72	2172	0.85	A/B
1M10_A_B	Medium	Von Willebrand Factor dom. A1	Glycoprotein IB-alpha	2.1	2097	0.65	E
1MAH_A_F	RB	Acetylcholinesterase	Fasciculin	0.61	2145	0.73	E
1ML0_AA_D	RB	Viral chemokine binding p. M3	Chemokine Mcp1	1.02	2069	0.57	O
1MLC_AB_E	RB	Fab44.1	HEW lysozyme	0.6	1392	0.96	A/B
1N2C_ABCD_EF	Medium	Nitrogenase Mo-Fe protein	Nitrogenase Fe protein	2.13	3635	0.59	O
1N8O_ABC_E	RB	Chymotrypsin	Ecotin	0.94	1851	0.88	E
1NCA_HL_N	RB	Fab	Flu virus neuraminidase N9	0.24	1953	0.92	A/B
1NSN_HL_S	RB	Fab N10	Staphylococcal nuclease	0.35	1776	0.86	A/B
1NW9_B_A	Medium	Capase-9	Ecotin	1.97	2112	0.75	E
1OPH_A_B	RB	Alpha-1-antitrypsin	Trypsinogen	1.21	1360	0.91	E
1PPE_E_I	RB	Bovine trypsin	CMTI-1 squash inhibitor	0.44	1688	0.77	E
1PXV_A_C	Difficult	Cystein protease	Cystein protease	2.63	2336	0.61	E

			inhibitor				
1QA9_A_B	RB	CD2	CD58	0.73	1353	0.63	O
1QFW_HL_AB	RB	Fv	Human chorionic gonadotropin	1.31	1580	0.91	A/B
1QFW_IM_AB	RB	Fv	Human chorionic gonadotropin	0.73	1637	0.77	A/B
1R0R_E_I	RB	Subtilisin Carlsberg	OMTKY	0.45	1409	0.76	E
1R8S_A_E	Difficult	Arf1 GTPase	Sec 7 domain	3.73	2986	0.64	O
1RLB_ABCD_E	RB	Transthyretin	Retinol binding protein	0.66	1439	0.73	O
1S1Q_A_B	RB	UEV domain	Ubiquitin	0.98	1288	0.61	O
1SBB_A_B	RB	T-cell receptor beta	Staphylococcus enterotoxin B	0.37	1064	0.71	O
1T6B_X_Y	RB	Anthrax protective antigen	Anthrax toxin receptor	0.62	1948	0.83	O
1TMQ_A_B	RB	alpha-amylase	RAGI inhibitor	0.86	2401	0.78	E
1UDI_E_I	RB	Uracyl-DNA glycosylase	Glycosylase inhibitor	0.9	2022	0.59	E
1VFB_AB_C	RB	Fv D1.3	HEW lysozyme	1.02	1383	0.85	A/B
1WEJ_HL_F	RB	Fab E8	Cytochrome C	0.31	1177	0.93	A/B
1WQ1_R_G	Medium	Ras GTPase	Ras GAP	1.16	2913	0.74	O
1XD3_A_B	RB	UCH-L3	Ubiquitin	1.24	2281	0.63	O
1XQS_A_C	Medium	HspBP1	Hsp70 ATPase domain	1.77	2350	0.68	O
1Y64_A_B	Difficult	Actin	BNI1 protein	4.69	2745	0.53	O
1YVB_A_I	RB	Falcipain 2	Cystatin	0.51	1743	0.75	E
1Z0K_A_B	RB	Rab4A GTPase	RAB4 binding domain of Rabenosyn	0.53	1787	0.62	O
1Z5Y_D_E	RB	N-term of DsbD	E.coli CCMG protein	1.23	1346	0.77	O
1ZHI_A_B	RB	BAH domain of Orc1	Sir Orc-interaction domain	0.68	1322	0.62	O
2AJF_A_E	RB	ACE2	SARS spike protein receptor binding domain	0.65	1704	0.70	O
2B42_B_A	RB	Xylanase	Xylanase inhibitor	0.72	2520	0.63	E
2BTF_A_P	RB	Actin	Profilin	0.75	2063	0.58	O

2C0L_A_B	Difficult	PTS1 and TRP region of PEX5	SCP2	2.62	2013	0.55	O
2CFH_A_C	Medium	BET3	TPC6	1.55	2384	0.54	O
2FD6_HL_U	RB	Plasminogen receptor antibody	Plasminogen activator receptor	1.07	1139	0.94	A/B
2H7V_A_C	Medium	Rac GTPase	YpkA	1.63	1574	0.73	O
2HLE_A_B	RB	Ephrin B4 receptor	Ephrin B2 ectodomain	1.4	2116	0.54	O
2HMI_CD_AB	Difficult	Fab 28	HIV1 reverse transcriptase	2.26	1234	0.96	A/B
2HQS_A_H	RB	TolB	Pal	1.14	2333	0.80	O
2HRK_A_B	Medium	Glutamyl-t-RNA synthetase	GU-4 nucleic binding protein	2.03	1595	0.83	O
2I25_N_L	RB	Shark single domain antigen receptor	Lysozyme	1.21	1425	0.91	A/B
2JEL_HL_P	RB	Fab Jel42	HPr	0.17	1501	0.89	A/B
2MTA_HL_A	RB	Methylamine dehydrogenase	Amicyanin	0.41	1461	0.77	E
2NZ8_A_B	Medium	Rac GTPase	DH/PH domain of TRIO	2.13	2599	0.69	O
2O8V_A_B	RB	PAPS reductase	Thioredoxin	1.37	1619	0.68	E
2OOB_A_B	RB	Ubiquitin ligase	Ubiquitin	0.85	808	0.40	O
2OT3_B_A	Difficult	Rab21 GTPase	Rabex-5 VPS9 domain	2.79	2306	0.67	O
2PCC_A_B	RB	Cyt C peroxidase	Cytochrome C	0.39	1141	0.64	E
2SIC_E_I	RB	Subtilisin	Streptomyces subtilisin inhibitor	0.36	1617	0.85	E
2SNI_E_I	RB	Subtilisin	Chymotrypsin inhibitor 2	0.35	1628	0.86	E
2UUY_A_B	RB	Trypsin	Tryptase inhibitor from tick	0.43	1280	0.59	E
2VIS_AB_C	RB	Fab	Flu virus hemagglutinin	0.8	1296	0.96	A/B
7CEI_A_B	RB	Colicin E7 nuclease	Im7 immunity protein	0.7	1384	0.77	E
